# Impact of using artificial intelligence as a second reader in breast screening including arbitration

**DOI:** 10.1038/s43018-026-01128-z

**Published:** 2026-03-10

**Authors:** Lucy M. Warren, Jenny Venton, Kenneth C. Young, Mark Halling-Brown, Christopher J. Kelly, Marc Wilson, Megumi Morigami, Lisanne Khoo, Deborah Cunningham, Richard Sidebottom, Mamatha Reddy, Hema Purushothaman, Delara Khodabakhshi, Lesley Honeyfield, Amandeep Hujan, Tsvetina Stoycheva, Andy Joiner, Reena Chopra, Aminata Sy, Dominic Ward, Lin Yang, Rory Sayres, Daniel Golden, Namrata Malhotra, Rachita Mallya, Lihong Xi, Della Ogunleye, Charlotte Purdy, Alistair Mackenzie, Susan Thomas, Shravya Shetty, Fiona J. Gilbert, Ara Darzi, Hutan Ashrafian

**Affiliations:** 1 Royal Surrey NHS Foundation Trust, Guildford, UK; 2 University of Surrey, Guildford, UK; 3 Google Research, Mountain View, CA USA; 4 St George’s University Hospitals NHS Foundation Trust, London, UK; 5 Imperial College Healthcare NHS Trust, London, UK; 6 The Royal Marsden NHS Foundation Trust, London, UK; 7 Imperial College London, London, UK; 8 AIMS Public Engagement Group, London, UK; 9 Google for Health, Mountain View, CA USA; 10 University of Cambridge, Cambridge, UK

**Keywords:** Breast cancer, Machine learning, Radiography, Cancer

## Abstract

The impact of incorporating artificial intelligence (AI) into a double-read breast-screening workflow, including arbitration, is unclear. This retrospective study included 50,000 representative women from two NHS breast-screening centers. All the women had long-term follow-up, allowing us to determine whether use of AI leads to earlier cancer detection. Cases requiring arbitration (8,732 cases) were read by 22 readers in a reader study, following their normal arbitration workflow. Overall, after arbitration, replacing the second reader with AI was noninferior (5% margin) to two human readers in terms of sensitivity and specificity (*P* < 0.001) while offering a workload benefit. Arbitration improved the specificity of the AI arm by overruling cases incorrectly recalled by the AI tool; however, it also overruled the AI tool recall decision for some interval and next-round cancers. Further development of the AI tool alongside improvement in its explainability could lead to the earlier detection of cancers.

## Abstract (web summary)

Warren et al. used data from a retrospective cohort of 50,000 women attending breast screening. Arbitration between human and AI decisions was performed in a reader study following normal arbitration workflow. After arbitration, replacing the second human reader with AI in a double-read breast screening workflow was noninferior to two human readers.

## Main

Breast screening aims to find cancers early, where treatment is more successful. The NHS Breast Screening Programme (NHSBSP) invites women aged between 50 years and 70 years for mammographic screening every 3 years, or women aged >70 years can self-refer. Each mammogram is read by two mammography readers (radiologists, breast clinicians, associate specialists, consultant radiographers or enhanced-level practitioners), who decide whether to recall the woman for additional follow-up. An arbitration process takes place to come to a final decision about whether to recall a woman, either if there is disagreement between the two mammography readers or for all recalled women, depending on local screening center processes. A radiologist workforce crisis in the UK threatens the long-term sustainability of the NHSBSP. The UK has a 30% shortfall of clinical radiologists and this is forecast to rise to 40% by 2028^1^. Artificial intelligence (AI)-assisted screening could play an important role in future proofing of the UK’s NHSBSP.

AI has shown promise as a standalone tool for detecting breast cancer in breast screening. It has the potential to reduce mammography reader workload while potentially improving outcomes. AI could be used in breast screening in several ways: (1) as a standalone tool to replace one of the human readers; (2) as decision support for readers; (3) as a triage tool by using the AI estimate of cancer likelihood to reduce screening by human readers for low-risk cases and increase it for high-risk cases; or a combination of these approaches^2^.

A systematic review^3^ of standalone AI performance for breast cancer detection at screening concluded that it performed as well or better than radiologists. However, it is less clear how radiologists perform when interacting with AI tools in situations closer to real-world screening. An important aspect of breast screening in the UK is the use of double reading with arbitration. A pilot of an independent external validation process^4^ concluded that there needed to be a clinical validation of the impact of AI on the decisions made by radiologists during arbitration. This study addresses this issue, by performing a large retrospective study including mammograms and clinical data from 50,000 women at two screening centers. There have been retrospective studies that have simulated the arbitration outcome with AI^2,5–7^. In addition, a small reader study (278 cases)^8^ looked at the impact on arbitration; however, this was not clinically relevant to the NHSBSP due to enriched dataset, use of ultrasound and mammography and different arbitration processes. The study presented here is a large-scale study using retrospective data, to perform a reader study with radiologists and consultant radiographers prospectively arbitrating studies, using arbitration protocols that are relevant to the NHSBSP.

The AI system used in this evaluation was created by Google (v1.2, Google LLC), and is an updated version of the v1.0 model^9^. Further details of the AI model are given in Methods and the relevant paper^10^. In the human arm of the study, the workflow is based on the recall decision of the historical two mammography readers (referred to as first and second human readers). In the AI arm of the study, the workflow is based on the recall decision of the first historical human reader and the AI tool. To determine the impact of the AI tool on arbitration, the arbitration criteria at each center were applied. Any cases requiring arbitration were read by pairs of readers. The arbitration decisions were made in a reader study with 22 readers. As historical data are used, the reader study results did not impact clinical care of the patients.

**Fig. 1:**
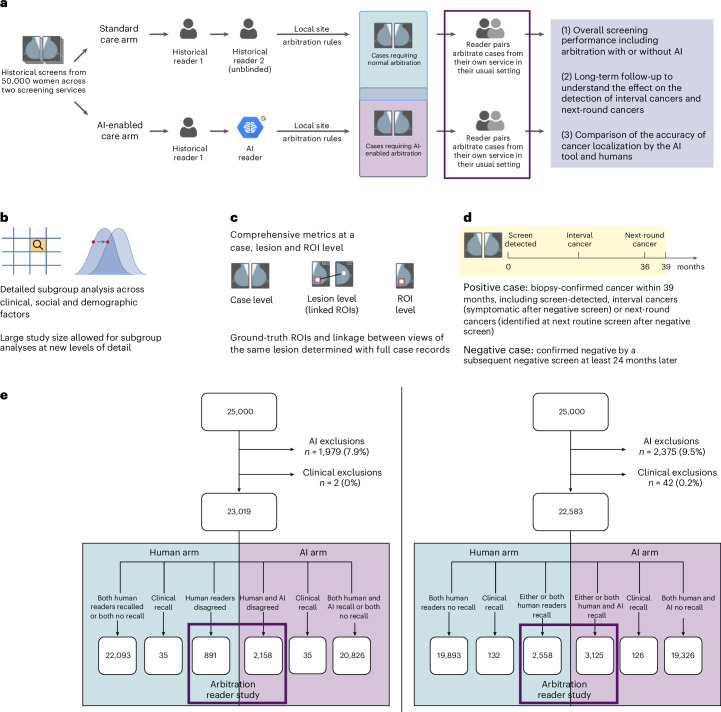
Overview of study design and aims. **a**–**e**, Study design (**a**), subgroup analysis (**b**), metrics (**c**), follow-up (**d**) and flowchart of data (**e**). Selection was for two clinical centers: center 1 (left) where discordant recalls are arbitrated and center 2 (right) where discordant and concordant recalls are arbitrated (*n* = 50,000 women). Source data

An overview of the study is given in Fig. 1. Some key strengths of the study include: the long-term (>3 years) follow-up enabled an assessment of whether an AI-assisted pathway can detect cancers earlier than standard care. The cancer locations and recall decisions after arbitration were annotated on the mammograms, allowing for the assessment of localization accuracy by the AI tool and humans, before and after arbitration. The study included two large screening centers, with different arbitration criteria. This allowed us to understand whether clinical pathway variability in current practice has an impact on cancer detection with AI. Finally, the scale of the study allowed for analysis by subgroups such as ethnicity, age, index of multiple deprivation, X-ray system manufacturer, cancer grade, invasive status and breast density.

## Results

### Study population

The study included mammography images and clinical data for 50,000 representative women from two NHSBSP screening centers in London (25,000 from each center) selected from the OPTIMAM Mammography Image Database OMI-DB^11^. Further details of the data selection are given in Methods. From the 50,000 women, 4,354 (8.7%) were excluded due to the AI exclusion criteria (technical recalls, cases containing more or less than four images and implants) and 44 (0.1%) cases were excluded due to insufficient or conflicting clinical information. After exclusions, 45,602 women were included. A flowchart of the data selection and overview of the study is shown in Fig. 1e. The distribution of age, ethnicity, X-ray system manufacturer and cancer grade are given in Table 1.

**Table 1:** Description of study population from each screening center

Characteristic	Center 1 (*n* = 23,019)			Center 2 (*n* = 22,583)		
Age, years						
Mean, years (s.d.)	58.7 (5.9)			58.7 (6.0)		
Range (years)	50.0–70.0			50.0–70.0		
50–54, *n* (%)	7,063 (30.7)			6,963 (30.8)		
55–59, *n* (%)	6,053 (26.3)			5,863 (26.0)		
60–64, *n* (%)	4,995 (21.7)			4,751 (21.0)		
65+, *n* (%)	4,908 (21.3)			5,006 (22.2)		
Ethnicity, *n* (%)^a^						
White	13,781 (59.9)			15,262 (67.6)		
Black	2,139 (9.3)			1,965 (8.7)		
Asian	4,274 (18.6)			1,990 (8.8)		
Mixed	646 (2.8)			460 (2.0)		
Other	1,021 (4.4)			608 (2.7)		
Not specified	1,158 (5.0)			2,298 (10.2)		
Manufacturer						
Hologic, *n* (%)	23,010 (100.0)			19,974 (88.4)		
GE Healthcare, *n* (%)	9 (0)			262 (1.2)		
Siemens, *n* (%)	0 (0)			2,347 (10.4)		
Clinical workflow						
Arbitration protocol	Discordant recalls			Discordant and concordant recalls		
Cancer type, *n*						
SD	169			183		
IC	68			68		
NSD	112			123		
Cancer grade	SD	IC	NSD	SD	IC	NSD
Invasive, *n*	138	58	87	144	53	93
G1	30	4	20	37	5	20
G2	86	20	52	67	26	52
G3	21	9	15	37	20	21
No grade	1	25	–	3	2	–
In situ	30	5	25	39	6	30
High	16	3	14	16	3	16
Intermediate	9	–	8	18	1	11
Low	5	–	3	2	1	3
No grade	–	2	–	3	1	–
Not specified	1	5	–	–	9	–

IC, interval cancer; NSD, next-round screen-detected cancer; SD, screen-detected cancer.

^a^Categories taken from the NHS Data Dictionary: https://www.datadictionary.nhs.uk/data_elements/ethnic_category.html.

### Sensitivity, specificity, recall rate and cancer detection rate

Table 2 shows the overall performance. The sensitivity and specificity in the AI arm were 1.2% (95% confidence interval (CI) −0.7%, 3.2%) and 0.3% (95% CI 0.0%, 0.6%) higher than the human arm, respectively. The sensitivity and specificity of the AI arm were noninferior (5% margin) to the human arm (*P* < 0.001). This satisfied the prespecified noninferiority endpoint. There was no significant difference in the recall rate between the AI arm and the human arm (AI versus human difference: −0.3% (95% CI −0.6%, −0.0%), *P* = 0.076). There was no significant difference in the cancer detection rate between the AI arm and the human arm (AI versus human difference 0.0% (95% CI −0.0%, 0.1%), *P* = 0.299).

**Table 2:** Overall performance metrics following arbitration

Performance metrics^a^	Center 1 (*n* = 23,019)				Center 2 (*n* = 22,583)				Both centers combined (*n* = 45,602)			
	Human am	AI am	Difference (%)	test^b^	Human arm	AI am	Difference (%)	test^b^	Human arm	AI arm	Difference (%)	test^b^
Sensitivity (%)	48.1 (42.9, 53.4)	50.1 (44.9, 55.4)	2.0 (−1.1, 5.1)	Noninferior *P* = 0.0001	47.9 (42.8, 52.9)	48.4 (43.3, 53.5)	0.5 (−2.0, 3.0)	Noninferior *P* = 0.0001	48.0 (44.4, 51.6)	49.2 (45.6, 52.9)	1.2 (−0.7, 3.2)	Noninferior *P* < 0.0001
Specificity (%)	96.4 (96.1, 96.6)	97.0 (96.8, 97.3)	0.7 (0.3, 1.1)	Superior *P* = 0.006	96.6 (96.4, 96.8)	96.6 (96.3, 96.8)	0.0 (−0.4, 0.4)	Noninferior *P* < 0.0001	96.5 (96.3, 96.7)	96.8 (96.6, 97.0)	0.0 (0.0, 0.6)	Noninferior *P* < 0.0001
PPV (%)	17.0 (14.7, 19.3)	20.7 (18.1, 23.6)	3.7 (2.4, 5.2)	N/A	19.1 (16.8, 21.7)	19.1 (16.7, 21.8)	0.1 (−1.2, 1.4)	N/A	18.0 (16.3, 19.8)	20.0 (18.1, 21.8)	1.9 (1.0, 2.9)	N/A
NPV (%)	99.2 (99.1, 99.3)	99.2 (99.1, 99.3)	0.0 (−0.0, 0.1)	N/A	99.1 (99.0, 99.2)	99.1 (99.0, 99.2)	0.0 (−0.0, 0.1)	N/A	99.1 (99.1, 99.2)	99.2 (99.1, 99.2)	0.0 (−0.0, 0.1)	N/A
Recall rate	4.3 (4.0, 4.6)	3.7 (3.4, 3.9)	−0.6 (−1.0, −0.2)	Significant *P* = 0.009	4.1 (3.9, 4.4)	4.2 (3.9, 4.4)	0.0 (−0.4, 0.4)	Not significant *P* = 0.899	4.2 (4.0, 4.4)	3.9 (3.7, 4.1)	−0.3 (−0.6, −0.0)	Not significant *P* = 0.076
CDR	7.3 (6.2, 8.4)	7.6 (6.5, 8.7)	0.0 (−0.0, 0.1)	Not significant *P* = 0.288	7.9 (6.8, 9.1)	8.0 (6.9, 9.2)	0.0 (−0.0, 0.1)	Not significant *P* = 0.724	7.6 (6.8, 8.4)	7.8 (7.0, 8.6)	0.0 (−0.0, 0.1)	Not significant *P* = 0.299

^a^For sensitivity, positive predictive value (PPV), recall rate and cancer detection rate (CDR) the total number of positive cases used for the calculations is 349 for center 1, 374 for center 2 and 723 for both centers combined. The sensitivity is around 50% due to the long-term follow-up such that a positive case includes screen-detected cancers, before interval cancers and before next-round screen-detected cancers.

^b^For sensitivity and specificity a one-sided Wald’s noninferiority test for paired data is used to determine noninferiority for difference and Obuchowski’s extension of two-sided McNemar’s test for clustered data is used to determine superiority. For recall rate and CDR, Obuchowski’s extension of two-sided McNemar’s test for clustered data is used to determine whether the difference is significant. N/A, not available; NPV, negative predictive value.

### Workload metrics

The impact on workload was considered separately for each center because they had different arbitration protocols. Center 1 arbitrated discordant recalls only, whereas center 2 arbitrated all recall decisions (discordant and concordant). As seen in Table 3, at both centers, the number of human screen readings in the AI arm was 50% lower than in the human arm, due to AI replacing the second reader. In addition, AI excluded 4,354 (8.7% of cases), which would still need to be read by two human readers, resulting in an overall 46% lower number of screen readings in the AI arm. However, the number of arbitrations required in the AI arm was 142% and 22% higher than in the human arm, for center 1 and center 2, respectively. It should be noted that different professional groups may be performing these different tasks, but this can vary by screening center.

**Table 3:** Workload metrics following arbitration

Workload metrics	Human arm	AI arm	Difference (%)	Human arm	AI arm	Difference (%)	Human arm	AI arm	Difference (%)
No. of reads	46,038	23,019	−50	45,166	22,583	−50	91,204	45,602	−50
No. of arbitrations (rate)	891 (3.9)	2,158 (9.4)	+142	2,558 (11.3)	3,125 (13.8)	+22	3,449 (7.6)	5,283 (11.6)	+53
Difference in reading time^c^	N/A	N/A	−36	N/A	N/A	−44	N/A	N/A	−40

^c^Assuming that the arbitration read takes five times the overall reader time of a single screen read.

### Impact of arbitration

Arbitration decisions in the reader study were made by pairs of readers. They together determined a consensus opinion about whether or not the woman needed to be recalled for further assessment, drawing a region of interest (ROI) around the area that they recalled. This mimicked the arbitration process performed clinically. A detailed description of the arbitration process is described in Methods.

**Fig. 2:**
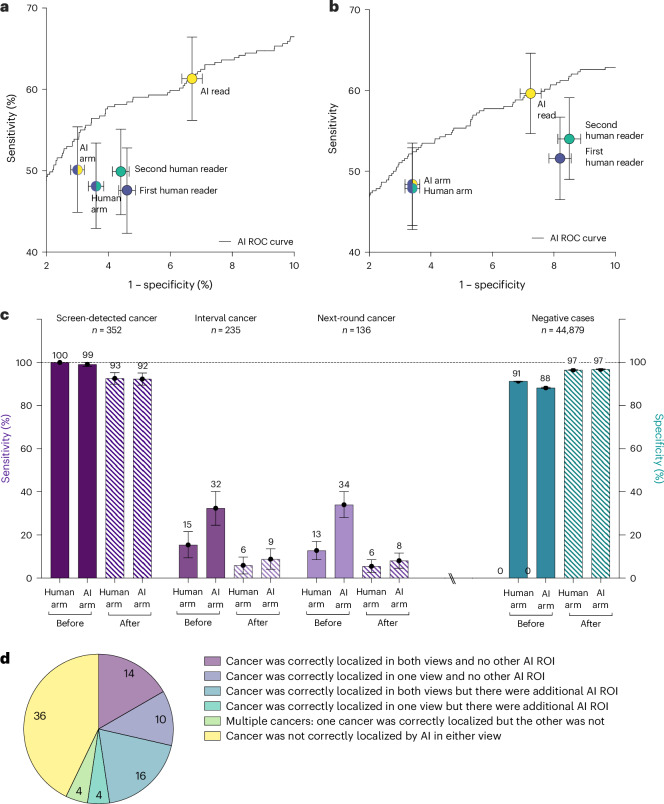
Comparison of performance before and after arbitration. **a**,**b**, Sensitivity and false-positive rate (1 − specificity) of the AI read^10^ as ROC curve (line), at the operating point used in the study (yellow), first (blue) and second (turquoise) historical reader before arbitration and AI arm (blue and yellow) and human arm (blue and turquoise) after arbitration, for center 1 (**a**) (*n* = 23,019 women) and center 2 (**b**) (*n* = 22,583 women), respectively. Error bars for sensitivity and specificity values are Wald’s CI. **c**, Sensitivity by time of cancer detection before and after arbitration for the AI and human arms and specificity before and after arbitration for the human and AI arms, for both sites combined. Numbers on top of the bars are the sensitivity or specificity for that bar and error bars are Wald’s CI (*n* = 45,602 women). **d**, Outcome of comparing the ROIs shown by AI at the ground-truth location, for the 84 positive cases that AI correctly recalled on a case level before arbitration, but the reader pair overruled at arbitration (*n* = 84 women). Source data

Before arbitration the AI tool had similar sensitivity and specificity at both centers (Fig. 2a,b). The sensitivity and specificity of the first and second human readers differed between the two centers before arbitration due to their different arbitration practices. After arbitration the difference between the two centers disappeared.

The sensitivity after arbitration was 48.0% in the human arm and 49.2% in the AI arm (Table 2). This may appear low, but a sensitivity of around 50% is expected, because of the long period of follow-up (39 months) used. This means that, in addition to screen-detected cancers, positive cases include interval cancers (symptomatic after negative screen) and cancers detected at the next screening round (3 years later). These would not have been detected by human readers at screening. We included the next-round cancers and interval cancers to assess whether the AI tool detects these cancers earlier than humans. The AI arm sensitivity was 92.3% for screen-detected cancers, 8.8% for interval cancers and 8.1% for next-round cancers (Table 4).

**Table 4:** Sensitivity and specificity before and after arbitration

	Center 1					Center 2					Both centers				
	*n*	Human arm		AI arm		*n*	Human arm		AI arm		*n*	Human arm		AI arm	
		Before arbitration (%)	After arbitration (%)	Before arbitration (%)	After arbitration (%)		Before arbitration	After arbitration (%)	Before arbitration (%)	After arbitration (%)		Before arbitration (%)	After arbitration (%)	Before arbitration (%)	After arbitration
Sensitivity															
Screen detected	169	100.0 (100.0, 100.0)	95.3 (92.1, 98.5)	98.8 (97.2, 100.4)	94.1 (90.5, 97.6)	183	100.0 (100.0, 100.0)	90.2 (85.8, 94.5)	99.5 (98.4, 100.5)	90.7 (86.5, 94.9)	352	100.0 (100.0, 100.0)	92.6 (89.9, 95.3)	99.1 (98.2, 100.1)	92.3 (89.5, 95.1)
Interval cancer	68	13.2 (5.2, 21.3)	4.4 (−0.5, 9.3)	32.4 (21.2, 43.5)	10.3 (3.1, 17.5)	68	17.6 (8.6, 26.7)	7.4% (1.1, 13.6)	32.4 (21.2, 43.5)	7.4 (1.1, 13.6)	136	15.4 (9.4, 21.5)	5.9 (1.9, 9.8)	32.4 (24.5, 40.2)	8.8 (4.1, 13.6)
Next-round cancer	112	11.6 (5.7, 17.5)	3.6 (0.1, 7.0)	34.8 (26.0, 43.6)	8.0 (3.0, 13.1)	123	13.8 (7.7, 19.9)	7.3 (2.7, 11.9)	33.3 (25.0, 41.7)	8.1 (3.3, 13.0)	235	12.8 (8.5, 17.0)	5.5 (2.6, 8.5)	34.0 (28.0, 40.1)	8.1 (4.6, 11.6)
Total^a^	349	54.7 (49.5, 60.0)	48.1 (42.9, 53.4)	65.3 (60.3, 70.3)	50.1 (44.9, 55.4)	374	56.7 (51.7, 61.7)	47.9 (42.8, 52.9)	65.5 (60.7, 70.3)	48.4 (43.3, 53.5)	723	55.7 (52.1, 59.4)	48.0 (44.4, 51.6)	65.4 (62.0, 68.9)	49.2 (45.6, 52.9)
Specificity															
Total	22,670	93.6 (93.3, 93.9)	96.4 (96.1, 96.6)	89.9 (89.5, 90.3)	97.0 (96.8, 97.3)	22,208	88.8 (88.4, 89.3)	96.6 (96.4, 96.8)	86.5 (86.1, 87.0)	96.6 (96.3, 96.8)	44,879	91.3 (91.0, 91.5)	96.5 (96.3, 96.7)	88.2 (87.9, 88.5)	96.8 (96.6, 97.0)

Values in brackets show the 95% CI. For sensitivity this is given for each type of positive case. For before arbitration, recall by at least one reader was a recall outcome. The *n* is the number of positive or negative cases for calculation of sensitivity and specificity.

^a^The total sensitivity is around 50% due to the long-term follow-up such that a positive case includes screen-detected cancer, before interval cancers and before next-round screen-detected cancers.

Figure 2c and Table 4 compare the sensitivity and specificity before and after arbitration. Before arbitration, recall by either reader was considered a recall decision for the case. Arbitration aims to improve specificity with minimal loss in sensitivity. Therefore, as expected there is a higher specificity and lower sensitivity after arbitration, in both arms. However, the difference in sensitivity before and after arbitration differs by time of detection. Before arbitration, the sensitivity of interval and next-round cancer was higher in the AI arm (32.4% and 34.0%, respectively), compared to the human arm (15.4% and 12.8%, respectively). However, after arbitration, the sensitivity of interval and next-round cancer are similar in the AI arm (8.8% and 8.1%) and the human arm (5.9% and 5.5%, respectively).

### Understanding cases that are overruled at arbitration

There were 93 positive cases that AI correctly recalled at a case level, but the reader pair overruled at arbitration. Of these 13 are screen-detected cancers, 28 interval cancers and 52 next-round cancers.

Most (94.5%) of the positive cases in the study had the ground-truth location annotated by expert radiologists or consultant radiographers who did not participate in the study. To understand the drop in sensitivity during arbitration, we reviewed these 93 positive cases that AI correctly recalled on a case level, but the reader pair overruled at arbitration.

We compared the ROIs drawn at arbitration to the ground-truth location. For 9 women there was no ground-truth location annotated, leaving 84 women. As shown in Fig. 2d, for 24 women the cancer was correctly localized in either one or both views and there were no other AI boxes. For 20 women the cancer was localized in at least one view but there were other AI boxes. For four women with more than one cancer, one cancer was correctly localized by the AI tool and the other was not. For 36 women, the cancer was not correctly localized by the AI tool. The arbitration readers were ‘very dissatisfied’ or ‘somewhat dissatisfied’ with AI’s assessment for 87% of these 93 cases, compared with 48% of AI arm-arbitrated cases overall. Furthermore, 75.3% of these 93 cases had prior images, compared with 50.5% for cases that went to arbitration in the AI arm of the study. Of these cases with prior images, readers said that the priors changed their decision to recall or not for 65.7% of cases, compared with 60.0% of all cases with priors that went to arbitration in the AI arm.

### Subgroups

**Fig. 3:**
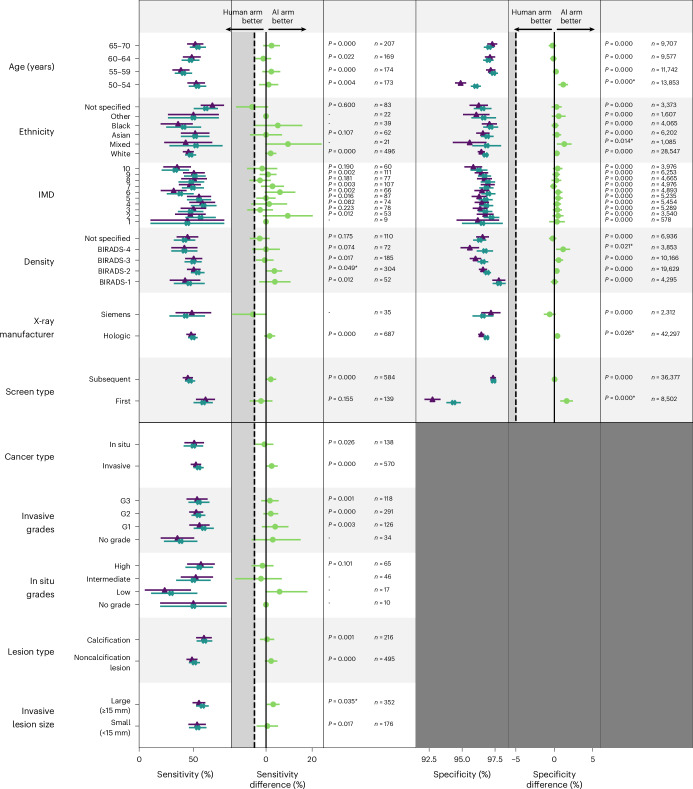
Performance by subgroup. Top: sensitivity and specificity for human arm (purple triangle) and AI arm (turquoise cross) with CIs, and difference in sensitivity and specificity (green circle) between arms for each of the subgroups for age, ethnicity, IMD density, X-ray manufacturer and screen type (*n* = 45,602 women). Bottom: sensitivity for human arm (purple triangle) and AI arm (turquoise cross) and difference in sensitivity (green circle) between arms for each of the subgroups for cancer type, invasive grades, in situ grades, lesion type and invasive lesion size. All characteristics are from the time of diagnosis of the cancer. Error bars for sensitivity and specificity values are Wald’s CI for groups with >50 cases and bootstrapped for groups with <50 cases. Data for differences are presented as the midpoint, lower and upper value of Wald’s interval for paired binary data. The *P* values with an asterisk (*) indicate a value <0.05 for AI arm superiority compared to the human arm, determined using Obuchowski’s extension of the two-sided McNemar’s test for clustered data. All other *P* values are for AI arm noninferiority compared to the human arm using Wald’s noninferiority test for paired binary data. The gray area in the difference column is the region below the 5% noninferiority margin. Adjustment for multiple comparisons was not performed and there is no defined level of significance for any *P* values (*n* = 724 lesions from 682 women; positive cases with both NBSS data and expert radiologist annotation data). Subgroups where the characteristic is not specified in the National Breast Screening System (NBSS), or subgroups with one case for sensitivity, are not displayed in this figure for clarity, but are included in the source data. Source data

The sensitivity and specificity of the two arms were calculated for each of the subgroups: X-ray system manufacturer, breast density category, age, type of screen (first or subsequent), index of multiple deprivation (IMD) and ethnicity (Fig. 3). We observed no notable differences in performance between the AI arm and human arm across the subgroups tested. Two subgroups where the AI arm sensitivity was lower than the human arm were: Siemens (AI versus human difference: −5.7 (95% CI −14.3, 0), *n* = 35), and ‘not specified’ ethnicity (AI versus human difference: −6.0 (95% CI −12.7, 0.6), *n* = 83). All were small groups, with large error bars, limiting the strength of statistical conclusions possible. For 25 out of 29 subgroups, the sample size was <300 positive cases, affecting the accuracy of sensitivity calculations.

Sensitivity was also calculated for each cancer subgroup: type, grade, lesion type and size (Fig. 3). We observed no notable differences in performance between the AI arm and the human arm across the subgroups tested. When split by subgroups, the numbers in the subgroups are low (for 11 out of 14 subgroups the sample included <300 positive cases) and in some subgroups there were <20.

### Localization performance

**Fig. 4:**
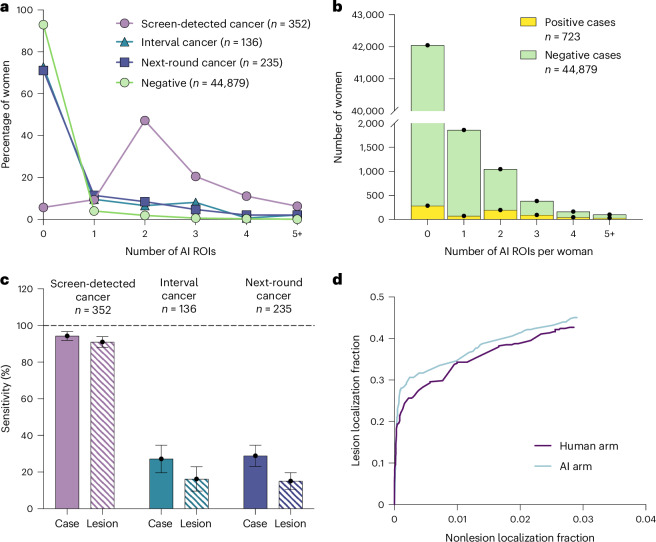
Localization analysis. **a**, The percentage of women with each number of AI ROIs for screen-detected cancers, interval cancers, next-round screen-detected cancers and negative cases, respectively (*n* = 45,602 women). **b**, The number of AI ROIs per woman for positive and negative cases that had at least one ROI (*n* = 45,602 women). **c**, Sensitivity before arbitration for the AI tool by time of cancer detection for centers 1 and 2 on case level and lesion level (using AI ROI). Error bars for sensitivity values are Wald’s CI for case level and lesion level. **d**, FROC for the AI arm and human arm for center 2 only where all recalled cases are arbitrated, so ROI available for analysis (using ROI drawn at arbitration; *n* = 723 women). Source data

The proportions of cases with varying numbers of AI-generated suspicious ROIs across the four images in each case are shown in Fig. 4a. Most negative cases have no ROI (93.0%) and the number of ROIs ranged from one to nine. For cases with screen-detected cancer, 94.3% of cases had at least one ROI, most had two ROIs and the number of ROIs ranged from one to ten. Interval cancers and next-round cancers had a distribution of ROIs more similar to the negative cases than screen-detected cancers, with 71.7% of cases having no ROI. Of those negative cases with ROIs, most had one ROI (Fig. 4b).

The sensitivity of the AI tool before arbitration by the time of cancer detection, at case and lesion levels is given in Fig. 4c. At a case level, the sensitivity of the AI tool is 27% and 29% for interval and next-round cancers and at a lesion level this reduces to 16% and 15%, respectively. This drop in sensitivity for lesion level compared to case-level analysis indicates that the AI tool is marking ROIs in the incorrect location for around half of such cases and highlights the importance of performing localization analysis in studies. The false-positive AI ROI rate was 0.12 per case for all cases, 0.12 per case for negative cases and 0.52 per case for positive cases. The false-positive AI ROI rate was 1.27 per case for the positive cases that the AI correctly recalled on a case level, but the reader pair overruled at arbitration.

The JAFROC figure of merit was 71.4 (95% CI 68.8, 74.0) for the AI arm and 70.5 (67.9, 73.1) for the human arm. The difference in the figure of merit was 0.88 (−0.63, 2.38) which was not statistically significant (*P* = 0.255). Figure 4d shows the free response operating characteristic (FROC) curve.

### Human factor analysis

In the post-study surveys completed by 21 of 22 readers, by the end of the study, most users reported that they ‘somewhat trusted’ the information provided by the tool on a 5-point scale of ‘did not trust at all’ (1 of 21), ‘somewhat trusted’ (15 of 21), ‘moderately trusted’ (4 of 21), ‘very trusted’ (1 of 21) and ‘extremely trusted’ (0 of 21). In an open-ended question asking in what situations did they think the AI was unreliable, if at all, over half the participants (12 of 21) stated that they thought that the AI was unreliable due to overcalling calcifications and for cases with prior images (12 for each statement, with 6 participants stating both statements).

## Discussion

There are a growing number of studies assessing the performance of AI compared to historical human reads in breast cancer screening using retrospective data^12–16^. However, such studies do not take into account arbitration. Some retrospective studies have simulated arbitration^2,5–7^; however, these cannot fully predict how human reader opinions will be impacted at arbitration when considering both human and AI opinions to come to a consensus. Here a reader study assessed the impact of replacing the second reader with AI, including arbitration.

Overall, on a case level, after arbitration, replacing the second reader with an AI read (AI arm) was noninferior for sensitivity and specificity, compared to two human readers (human arm). The AI arm had a slightly higher cancer detection rate and a slightly lower recall rate than the human arm, but these differences were not statistically significant.

There are seven aspects that we considered and discuss in this section. First, we considered what the workload implications are of replacing a second reader with AI. The human reading workload at screening in the AI arm was 46% lower than in the human arm because the AI tool replaced the second reader. However, the arbitration rate in the AI arm compared to the human arm increased from 3.9% to 9.4% at center 1 and from 11.3% to 13.8% at center 2. These increases, although different between centers due to the different arbitration approaches, are broadly in line with the simulations of arbitration in previous studies^6,7^. In addition, 8.7% of cases were not read by the AI tool, due to being within the AI tool’s exclusion criteria. These cases would need to be read by two human readers if the tool were to be used clinically. Therefore the resources for this and the infrastructure to facilitate this different workflow would need to be considered before deployment. Taking this all into account, the overall reductions in reading time for centers 1 and 2 were 36% and 44%, respectively, using a simplified assumption that an arbitration read takes about 5× as long as a single read^17^. A full cost analysis was not performed here. However, such an analysis using information from this study could be useful for planning prospective trials and the introduction of AI into the NHSBSP.

Second, we considered whether replacing a second reader with AI leads to breast cancers being detected earlier. A strength of this study is the longitudinal nature of the retrospective data, which allows us to assess performance by time of detection of positive cases. Before arbitration, the AI tool had similar sensitivity to first and second human readers for screen-detected cancers and a higher sensitivity than first and second human readers for interval cancers and next-round cancers. This implied that the AI tool could detect cancers earlier than two humans and we investigated whether this prevailed through arbitration. Arbitration improved specificity at both centers in both arms. For screen-detected cancers, there was a small decrease in sensitivity after arbitration in both arms. For interval cancers and next-round cancers, there was a larger reduction in sensitivity after arbitration and the decrease was larger for the AI arm than the human arm. Consequently, there was not a notable difference in sensitivity between the two arms for interval cancers and next-round cancers after arbitration. Therefore, after arbitration, replacing the second reader with AI did not result in cancers being detected earlier.

Third, we considered why some correct AI prompts were dismissed at arbitration. Arbitration here succeeded in improving specificity with minimal reduction in sensitivity. However, there were some cases (93 out of 8,732 arbitrations) that were correctly recalled by the AI tool, but were overruled at arbitration. These were primarily (86%) interval cancers and next-round cancers and therefore challenging cases not recalled clinically. For half the cases, the AI tool correctly localized the cancer (albeit half of these also had other ROIs in incorrect areas). For the other half of the cases, the AI tool did not correctly localize the cancer, which would explain why the AI tool was overruled. A higher proportion of these cases had prior images (75.3%), compared with all arbitrated cases (48.4%) and the readers indicated that the prior changed their decision 71% of the time. Therefore, it could be that some were overruled due to the readers seeing no change in the mammograms between the current and prior image and/or knowing that the AI tool does not analyze the prior images. Or perhaps the issue is more fundamental and readers cannot see why recall recommendations are being made by the AI tool.

The false-positive AI ROI rate was 0.12 per case for negative cases. This is much better than in traditional computer-aided diagnosis, for instance a previous prospective study in the UK NHSBSP^17^ found a false-positive AI ROI rate of 1.59 per case. Here for positive cases the false-positive AI ROI rate was 0.52 per case. On the positive cases that were correctly recalled by the AI tool, but were overruled at arbitration, there were nearly 3× more false-positive ROIs than for positive cases where human arbitration agreed with the AI decision to recall. These superfluous ROIs may be a contributing factor to the readers overruling the AI tool for some positive cases.

These 93 incorrectly overruled cases need to be weighed against the 2,307 out of 3,124 (73.8%) times that the human arbitration correctly overruled the AI tool when it recalled a negative case. It should also be noted that 8,148 of the 8,732 arbitrations were negative cases, highlighting the challenge that arbitration readers faced to identify the true positive cases among such large numbers of arbitration reads. Therefore, arbitration is a very challenging task for the readers, but they did a very good job of improving specificity with a small sensitivity loss, which is the aim of arbitration.

Fourth, we considered whether these results change with different subgroups. Sensitivity was analyzed across subgroups. The AI arm performed better than the human arm for Hologic images, but the opposite was true for Siemens images (although with a smaller number of cases). This was found before arbitration^10^ and persisted after arbitration (albeit with small numbers). Only 0.9% of the images used to train the AI tool were Siemens images. In the NHSBSP, the proportion of machines by manufacturer is 1% Fuji, 31% GE, 52% Hologic and 16% Siemens (J. Loveland, personal communication, National Co-ordinating Centre for the Physics of Mammography, 26 February 2025). The wide range of image processing used in the NHSBSP could also affect AI performance^18^. It has been shown that software upgrades by an X-ray vendor caused a change in AI performance^19^. This highlights the need to evaluate the performance of AI tools by X-ray system manufacturers and also to monitor the performance over time once deployed. There may also be a need to re-tune or re-train AI tools on deployment at a new site or after equipment upgrade.

Fifth, we considered whether the results differ by screening workflow. NHSBSP guidance recommends that breast-screening services should determine their local reading policy (Section 1.1.7 in https://www.gov.uk/government/publications/breast-screening-guidance-for-image-reading/breast-screening-guidance-for-image-reading). The two arbitration protocols here are the two main methods used in the NHSBSP and allowed the impact of an AI reader in different arbitration protocols to be examined. Before arbitration, the performance of readers at the two centers was very different due to the different clinical workflow used (Fig. 2a,b), whereas AI tool performance was very similar at both centers. This contributed to the larger increase in arbitrated cases with the introduction of AI at one center. After arbitration, the difference between the centers disappeared. Therefore, AI vendors should consider carefully the operating point or threshold of their tool at each screening center and how this complements the workflow and arbitration practice.

Sixth, we considered the strengths and limitations of the study design. The main strength of this study was the availability of long-term follow-up allowing us to investigate whether the AI arm could detect cancers earlier than the human arm. Having the locations of cancerous lesions, along with the AI ROI and the ROI drawn by readers at arbitration, allowed analysis at a lesion level. Although other studies have looked at localization accuracy^12,20^, our study compared localization accuracy at arbitration. The AI tool excluded 8.7% of women. The proportion of episode outcomes, age, ethnicity, IMD, X-ray system manufacturer and type of screen (first or subsequent) were not significantly different between the included and the excluded women. A limitation here is that, for 25.6% of women at center 1 and 7.5% of women at center 2, the paperwork used in the study had the historical human reader opinion, but no location data. If these 954 women were excluded from the study, the study results remain unchanged, that is, overall sensitivity and specificity remained noninferior. Ethnicity data were grouped according to categories taken from the NHS Data Model and Dictionary Service (https://digital.nhs.uk/services/nhs-data-model-and-dictionary-service#top). Ideally more detailed ethnicity subgroups should be analyzed, but the amount of data in each subgroup was too small. This is despite this study including 50,000 women overall and highlights the need for enriched datasets as well as representative datasets when investigating performance by ethnicity. This study used data from the NHSBSP where screening is performed 3 yearly. In other countries where screening is biennial or annual, there will be smaller numbers of interval cancers due to more frequent screening, which could impact the generalizability of these results to those programs. In this study, the same noninferiority margin was used for specificity and sensitivity as in other studies^6,21^. This did not impact the results because specificity was similar in both arms. However, due to the larger number of negative cases compared with positive cases, the study could have been powered for a smaller noninferiority margin in specificity. Having different noninferiority margins for sensitivity and specificity should be considered in future. It was not possible to blind the AI arm to the readers because the AI output was overlaid on images and the human readers’ decisions on paperwork. However, this is clinically realistic because it is how the images would be read clinically. This may introduce bias, but there will also be bias in a real-world setting. This bias may change over time, through additional training and daily experience with AI. Finally, by re-reading the standard care arm (human arm), rather than using historical clinical outcome as a comparator, both arms were read similarly, reducing any laboratory effect.

Finally, we considered whether there is a better way in which the AI could be used. Some interval cancer cases and cancers detected at the next screening round were recalled by AI, but were overruled at arbitration despite AI localizing the cancer correctly in around half of these. This may be overcome by displaying the type of cancer localized (mass, calcification), incorporating priors into model prediction, reducing false-positive prompts to prevent readers being distracted, more explainability in the model output and providing AI confidence values for each ROI.

Post-study surveys completed by 21 of 22 readers indicated that, as well as changes to the AI tool listed above, training of readers may help readers understand better when to trust the AI tool. Feedback about AI predictions and actual cancer outcomes may help readers interpret AI predictions better, especially around superfluous ROIs as seen in other AI-assisted reader settings^22^. The human factor post-study survey in this study was exploratory. Further work could include a more extensive questionnaire study design to assess participants’ views, including on wider workforce considerations.

In addition, the operating point or threshold of the AI tool could be altered. The operating point of the AI tool determines its sensitivity and specificity and therefore the arbitration rate. The prespecified operating point in this study was selected using a tuning dataset, based on set rules^10^, which struck a balance across sensitivity, specificity and arbitration rates. These rules were pre-agreed between the members of the research team. It is possible to construct a different set of rules that weigh the target metrics differently and select a different operating point that could be, for example, more specific, to reduce the number of arbitrations required. In a live screening deployment, the screening center staff would agree to the operating point selected.

Finally, there could be alternative ways in which the AI tool could be incorporated into screening. One alternative could include sending cases where the AI tool had very high confidence straight to recall, avoiding arbitration^15^ or adaptive screening, offering certain groups earlier follow-up^23^ or additional imaging^24^. At any rate, a more effective human AI interface is needed to optimize implementation of AI tools in breast screening. We hope that the lessons learnt from this study will help inform upcoming prospective clinical trials investigating deployment of AI tools in breast screening, such as the EDITH trial (https://www.gov.uk/government/news/world-leading-ai-trial-to-tackle-breast-cancer-launched).

In conclusion, this study explored the effect of introducing AI as a second reader in a double-reader workflow, crucially including the process of specialist arbitration. It showed that AI-enabled reading was noninferior to a standard two-reader workflow. It highlighted that, when replacing a second reader with AI, overall reading workload was reduced. Further development of the AI tool alongside improvement in explainability and acceptance of the tool by mammography readers could lead to the detection of cancers earlier than with two human readers.

## Methods

This study is part of the Artificial Intelligence in Mammography Screening (AIMS) study. The AIMS study protocol was approved by East Midlands Nottingham Research Ethics Committee (no. 22/EM/0038) and NHS England Breast Screening Programme Research Advisory Committee (no. BSPRAC_0093). The study was registered with the ISRCTN (no. 60839016). The AIMS study was funded by a National Institute for Health and Care Research (NIHR) award from the Secretary of State for Health and Social Care. An overview of the study is given in Fig. 1a–e. Further information on research design is available in the Nature Portfolio Reporting Summary linked to this article.

### Case selection

Mammography images and clinical data for 50,000 women from two NHSBSP screening centers were selected from the OPTIMAM Mammography Image Database OMI-DB^11^.

The AI developer had used 10,000 cases from each screening center to select the operating point or threshold that is optimal for the recall rate for the local center. The vendor advised that they would do this before clinical implementation at any NHSBSP screening centers and so this mimicked the clinical situation. Therefore, all episodes for these women from any year were removed before selection of the study dataset.

We randomly selected 25,000 women aged 50–70 years per screening center from 2016. This permitted a 3-year follow-up in 2019, to avoid any potential impact of COVID-19 on the data. At both screening centers, the interval cancers in the National Breast Screening System (NBSS) were lower than expected from the national Screening History Information Management system. Therefore, the additional interval cancers to reach the numbers reported by the Screening History Information Management system were selected from a wider year range: 2011–2018. There was a proportion of women with normal mammograms, but no subsequent normal mammogram to confirm the negative ground truth. To ensure a high-quality ground truth, women younger than 68 years with normal mammograms, but no subsequent screening episode, were replaced with women who did have a follow-up mammogram from a wider year range: 2011–2018. The women were matched by episode outcome, whether it was the first or the subsequent mammogram, and by age (for screening center 1 within ±1 year and for screening center 2 within ±3 years). Women aged 68+ years were permitted to have no follow-up screen, because they would not typically be invited back as part of national screening at this age. For screening center 2, there was also a proportion of women whose cases had been used to train the AI tool. These were also replaced with women who had not been used to train the AI tool, with the same matching criteria as above.

The clinical data included pathological information and the recall or no-recall decisions by the historical first and second readers and arbitration. The locations of cancers for 94.5% of positive cases were recorded with a rectangle around the cancer or area where the cancer was later detected for those detected as interval cancers or at the next screening round. These bounding boxes were the ground-truth ROIs. For the remaining 5.5% of positive cases there were insufficient clinical data available for such annotation.

AI exclusion criteria were applied for technical recalls, any study containing >4 images or <4 images, or cases with implants, resulting in 4,354 women (8.7%) being excluded. The AI tool was run on the mammography images for the women not excluded and an AI recall decision for each woman was obtained using the site-specific operating points^10^. This included a case level decision and ROIs (bounding boxes marking suspicious areas). In addition, 44 (0.1%) cases were excluded due to insufficient or conflicting clinical information.

In the human arm of the study, the workflow was based on the recall decision of historical first and second human readers. In the AI arm of the study, the workflow was based on the recall decision of the first historical human reader and the AI tool. To determine the impact of the AI tool on arbitration, the arbitration criteria at each center were applied and selected cases read in a reader study with two readers making the arbitration decision. At center 1, women went to arbitration if, for either breast, there was a disagreement between the first and second readers. At center 2, women went to arbitration if recalled by either the first or second reader or both. A flowchart of the case selection, study exclusions and allocation to arms is given in Fig. 1e.

### Design of arbitration reader study

#### AI system

The AI system used in this evaluation was created by Google (v1.2, Google LLC) and is an updated version of the v1.0 model^9,10^. This is an AI-powered, independent mammography reader product for double-read breast cancer-screening workflows. It analyses two-dimensional, full-field digital mammograms to give a normal or abnormal screening determination and highlights suspicious ROIs. The AI system has three components: (1) a global model which takes four mammograms and produces a case-level prediction; (2) a detection model which detects bounding boxes of lesions for each view; and (3) a hybrid model which takes as input the features from the last layer of the global model and the bounding boxes from the detection model to produce a score for each bounding box. The final case-level cancer prediction is the maximum score of the bounding boxes for that case. The AI system outputs DICOM images with bounding boxes with scores above the operating point shown; however, case-level or bounding box scores are not displayed to the user. Data from 76,142 women (63,918 from the UK, 12,224 from the USA) were used to train the AI tool. Among all the studies, 88.7% were with Hologic images, 9.6% GE images, 0.9% Siemens images and 0.8% Philips images. The exclusion criteria of the AI tool include technical recalls, cases containing more or less than four images and implants. The four-image limitation is due to design of the AI tool where it processes one image for each of the four mammogram views (that is, left craniocaudal, left mediolateral oblique, right craniocaudal and right mediolateral oblique) for a complete analysis (no missing view allowed) and, when multiple images of the same view are present, it defers the selection of that image to the operator.

#### Readers

Nine radiologists from center 1 and nine radiologists and four consultant radiographers from center 2 participated in the reader study. All were NHSBSP-accredited mammography readers, with between 3 years and 36 years of experience (mean = 13.5 years) and reading between 2,300 and 15,000 examinations per year (mean = 6,000). Only one reader had experience in using AI previously.

#### Reader training

All readers were provided with an information pack and completed a consent form before the study. All readers completed training in interpreting the AI tool. This was provided by the AI vendor to mimic what would happen clinically and included a 10-min video explaining the AI tool and 100 cases that showed the AI decision and the ground truth—cancer or no cancer—and the location of any cancers. These training cases were from a screening center not included in the study. In addition a pilot study was performed by the research team. This included 28 cases, to train the readers in how to use the viewing software (RiViewer) and to test the entire process before the main study, including paperwork generation, hanging protocol, clarity of questions asked and timing. There was no overlap between the pilot study cases and the cases used in the main study.

#### Study paperwork

Clinically, when making arbitration decisions, the arbitration panel can view the decisions of the first and second readers on the NBSS and the clinical paperwork, where the readers have written their opinion and/or drawn areas of suspicion on a diagram. It was not possible to show the readers in this study the original paperwork or NBSS because this would show the original arbitration decision and the data would not be anonymized. To overcome this, research radiographers laboriously transcribed the original first and second reader opinions and diagrams of suspicious regions to create an anonymized copy of the study paperwork blinded to the screening outcome. For cases in the human arm, the study paperwork contained the opinions of both first and second readers. For cases in the AI arm, the study paperwork contained only the first human reader opinion (the second reader is AI).

#### Arbitration reading conditions and hanging protocol

Batches of ten cases for arbitration were reviewed by pairs of readers. As these sessions were outside of working hours, the pairing depended primarily on working patterns. This mimics the clinical situation, where readers working at the same time arbitrate together. The pairs were not fixed, to allow for flexibility around clinical and personal commitments. The pair reading each batch was recorded. The reading took place on clinical workstations at the screening center using RiViewer software in a reading room with normal clinical conditions, including low lighting and high-resolution monitors.

The proportion of AI arm and human arm cases within a batch was based on the proportion in the entire dataset at that center. The readers saw the study images (termed ‘current images’), the images of the immediately prior screening round if there was one and, for the AI arm, the images produced by the AI model with any areas of concern annotated. For both arms, the paperwork was shown after the readers had looked at the current images and prior images. For the AI arm, the study paperwork was shown at the same time as the AI images, so that, for images in the AI arm, the readers saw the human and AI decisions at the same time. For both arms, the readers had to complete a whole loop of a defined hanging protocol before they could make a decision for that case. It was not possible to blind the AI arm to the readers because the AI output was overlaid on images and the human readers’ decisions on paperwork. However, this is clinically realistic because it is how the images would be read clinically

For all cases readers were asked to provide the Royal College of Radiology 5-point scale M-score^25^ (M1, no recall; M2, no recall; M3, recall; M4, recall; and M5, recall) for each breast, and the breast density Breast Imaging Reporting and Data System (BIRAD) categories A–D. For cases with prior imaging, they were asked additionally whether the priors changed their recall decision. For cases in the AI arm, they were also asked whether they were satisfied with the AI assessment of the case. If recalling a case, the reading pairs were asked to draw a bounding box around the areas being recalled and provide the conspicuity, lesion type and suspicion of malignancy. The readers were asked to draw a rectangle around the region in both views. Each region has an ID and, if they saw it in both views, they linked the region with the same ID.

#### Data quality control

Collection of all clinical data and images was automated and the images and data fields were not altered during collection. This ensured that the data were clinically relevant and representative. The reader study required study paperwork to be transcribed from clinical paperwork by research radiographers. The trial manager checked that the clinical paperwork had been correctly transcribed for 1% of the study paperwork during on-site monitoring. The data entered by the readers from the reader study were checked fortnightly with automated scripts, for any inconsistencies or incomplete data. These data checks were outlined in a data management plan at the start of the study.

#### Exploratory human factor surveys

Participants completed online surveys before, during and after the study. This included relevant questions from the NASA Task Load Index^26^, trust and general impressions of the AI tool. Results in this paper are shown for the surveys after the study.

### Positive and negative definitions used in the analysis

#### Positive cases

A positive case is a woman diagnosed with cancer within 39 months of the screening mammogram used in the study, based on pathological information. This therefore includes screen-detected cancers, interval cancers and screen-detected cancers detected at the next screening round.

#### Negative cases

A negative case is a woman whose mammograms used in the study resulted in an outcome of normal, with routine recall to screening 3 years later and the follow-up mammograms from 24 months onward also resulted in an outcome of normal with routine recall to screening 3 years later (age <68 years only)

### Localization ground truth

The mammograms of all the positive cases were annotated by expert radiologists or consultant radiographers who did not participate in the study. They drew a rectangular ROI tightly around each lesion. They then described the radiological appearance of the lesion (mass, distortion, asymmetry, calcification), whether the lesion was malignant or benign and the conspicuity of the lesion on a three-point scale (very subtle, subtle or obvious). Conspicuity was defined as how visible the lesion was in the image, in the annotator’s judgment. For interval cancers and next-round cancers, the cancer was annotated on the diagnostic image (where available) and, in addition, the location the cancer would have been as annotated in the prior image.

### Statistics and reproducibility

#### Study characteristics

Descriptive analysis was used to summarize study population characteristics. Frequencies and percentages were calculated for categorical data. A *χ*^2^ test was used to compare proportions of characteristics between included and excluded groups.

#### Primary analysis

Our primary endpoint was noninferiority (prespecified 5% absolute margin) of the AI arm for sensitivity and specificity at the case level, compared to the human arm, measured against a 39-month ground truth. Statistical testing was performed using one-sided tests at the 0.025 significance level (after correcting for multiple comparisons using Holm–Bonferroni). CIs on the difference were Wald’s intervals^27^ and Wald’s test was used for noninferiority^28^. Both used Obuchowski’s variance estimate^29^. If noninferiority was shown, a one-tailed superiority test was planned to follow without loss of power or requirement for multiple testing^30,31^. Superiority comparisons were conducted using Obuchowski’s extension of the two-sided McNemar’s test for clustered data. Clusters were defined to group arbitrations read by the same reader pair. For case-level analysis the highest RCR M score for each breast was used. The data met the requirements of the paired binary tests used (Wald’s and McNemar’s tests).

#### Secondary analysis

Case-level secondary analysis included positive predictive value (PPV), negative predictive value (NPV), cancer detection rate (CDR) and recall rate (RR). For PPV and NPV, CIs on the absolute values, differences and CIs on difference were calculated by bootstrapping. For CDR and RR, differences were calculated using Obuchowski’s extension of the two-sided McNemar’s test for clustered data. For CDR and RR, Wald’s CIs were calculated with Obuchowski’s clusters based on reader pairs.

#### Exploratory analysis

Case-level subgroup sensitivity and specificity were calculated by type of screen, age, ethnicity, X-ray system manufacturer, IMD and breast density. In addition, subgroup sensitivity was calculated by cancer type, cancer grade, lesion characteristic and lesion size.

The age was taken from the NBSS. The grouping of age (50–54 years, 55–59 years, 60–64 years, 65–70 years) used as subgroups was as reported in published NHSBSP statistics. The ethnicity was taken from the NBSS. The grouping of ethnicities (white, mixed, Asian, black, other, not specified) as subgroups was based on the NHS Data Dictionary ethnic categories (https://www.datadictionary.nhs.uk/data_elements/ethnic_category.html). The IMD 1–10 (as defined in https://www.gov.uk/government/statistics/english-indices-of-deprivation-2019) was calculated from lower layer super output area data before de-identification. Breast density values (BIRADS 1–4) were calculated for mammograms acquired using Hologic devices with software developed by Royal Surrey^32^. The breast density subgroups were the categories from BIRADS, 5th edn. X-ray manufacturer values (Hologic and Siemens) were taken from the DICOM header of the mammography images. The screen type (first or subsequent screen) was taken from the NBSS. The subgroups used were as in NHSBSP statistics. The cancer type (invasive or in situ) was taken from the NBSS. These subgroups are reported in published NHSBSP statistics. The invasive grades (1, 2 and 3) and in situ grades (low, intermediate and high) were taken from the NBSS. The subgroups were based on the NHS Data Dictionary tumor grades for breast screening (https://archive.datadictionary.nhs.uk/DD%20Release%20June%202023/attributes/tumour_grade_for_breast_screening.html). The lesion type was obtained by an expert radiologist annotating the cancers; if that was not possible due to the diagnostic images not being available, the lesion type was taken from the NBSS. The invasive lesion size (small, <15 mm, and large, ≥15 mm) was taken from the NBSS. The subgroups used were as in NHSBSP screening statistics.

As the study was not powered for subgroup analysis and there were no prespecified subgroup endpoints, these subgroup analyses should be considered exploratory and hypothesis generating. We therefore present unadjusted CIs for subgroup differences to describe observed trends and magnitudes of effect within subgroups. It is important to note that these CIs should be interpreted cautiously due to the lack of power and the increased risk of false-positive findings associated with multiple subgroup comparisons. No formal hypothesis testing or multiplicity adjustments were conducted for these exploratory subgroup analyses. Case-level CIs were calculated using Wald’s CIs for groups of >50 cases and, for groups of <50 cases, bootstrapping was used.

Finally, localization analysis of the bounding boxes drawn during arbitration was performed using the RJafroc package v2.1.2 in RStudio v4.3.3 (ref. ^33^). A correctly localized lesion was defined as the overlap between the ROI drawn at arbitration and the corresponding ground-truth ROI having an intersection over union value ≥0.1. All intersections over union values <0.3 were reviewed by a radiologist who did not participate in the reader study and the hit-or-miss decision was changed accordingly.

For human factor analysis, perceived task load differences for the human and AI arm were analyzed using Wilcoxon’s signed-rank test. Other questions, such as those on trust and general impressions, were examined using descriptive statistics for closed-ended questions and open-ended responses underwent dual-coder thematic analysis.

#### Post-hoc analysis

For all positive cases where the AI correctly recalled but human arbitration then overrode the decision, we checked whether the AI ROI had correctly localized the ground-truth ROI. In addition, the average number of false-positive prompts per case were calculated for: all cases, positive cases, negative cases and positive cases where the AI correctly recalled but human arbitration then overrode the decision. For the positive cases, 2 × 2 tables of outcomes for the human and AI arms were provided for all positive cases (Supplementary Table 1), screen-detected cancers only (Supplementary Table 2) and negative cases only (Supplementary Table 3).

#### Sample size estimation

We powered the study by simulating a two-arm, within-case design (routine versus AI assisted), where each case is read under both regimens and the primary analysis is a matched-pair Wald’s test for noninferiority on sensitivity (specificity was expected to be amply powered, given the low prevalence). We assumed identical underlying performance in both arms: latent continuous scores with area under the curve of 0.90, binarized at a common threshold to yield 73% sensitivity and specificity using 39-month outcomes^9^. Between-arm correlation was modeled via an agreement parameter set to 84.5%, matching previously observed R1–R2 concordance on positives. We modeled the two site-specific arbitration protocols (R1 | R2 and R1 ≠ R2) and powered the study using a worst-case scenario that combined the R1 | R2 arbitration style, consensus panel recall of 0.73 and agreement between arms of 0.70. Under these assumptions, 275 cancer-positive cases exceeded 90% power, whereas 200 positives provided 80% power. We therefore targeted a minimum of 200 positive cases per site to achieve 80% power. Assuming a population prevalence of cancer, this corresponded to 25,000 cases per site.

#### Reproducibility

Randomization is not applicable to this study because it was a retrospective study and all clients were in both arms of the study. As described above, it was not possible to blind the AI arm to the readers because the AI output was overlaid on images and the human readers’ decisions on paperwork. However, this is clinically realistic because it is how the images would be read clinically. The data met the requirements of the paired binary tests used (Wald’s and McNemar’s tests). The data exclusions were defined before the study. From the 50,000 women, 4,354 (8.7%) were excluded due to being within the AI exclusion criteria (technical recalls, cases containing ≥4 or ≤4 images and implants) and 44 (0.1%) cases were excluded due to insufficient or conflicting clinical information.

### Reporting summary

Further information on research design is available in the Nature Portfolio Reporting Summary linked to this article.

## Supplementary information

Supplementary Information Protocol.

Reporting Summary

Supplementary Tables Supplementary Tables 1–4.

## Source data

Source Data Fig. 1 Data for Fig. 1e.

Source Data Fig. 2 Data for Fig. 2a–d.

Source Data Fig. 3 Data for Fig. 3.

Source Data Fig. 4 Data for Fig. 4a–d.

## Data Availability

The images and clinical data used in this publication are from the OPTIMAM imaging database and are not publicly available due to restrictions imposed by OPTIMAM’s ethical approval. Instead, the images and data can be accessed after a formal data access request and review by a Data Access Committee and a Data Sharing Agreement (DSA) implemented. Applications for access to the data can be made at https://medphys.royalsurrey.nhs.uk/omidb/getting-access/. The application, review and agreement process can take anywhere from 2 weeks to 12 weeks depending on the applicant’s desire to customize the template DSA. The dataset derived from this resource, which supports the primary findings of this study, is available in Supplementary Table 4. Source data are provided with this paper.

## Code availability

Statistical analysis and plotting were performed using open source and publicly available Python packages (numpy v1.24.4, pandas v2.1.1, scipy v1.9.1, matplotlib v3.7.2 and seaborn v0.13.0), using the methodology outlined in Methods. All codes were executed in Python (v3.9.7). The AI tool (v1.2, Google LLC) was not developed in this study; it was evaluated. The code used for training the models has a large number of dependencies on internal tooling, infrastructure and hardware, and its release is therefore not feasible. However, implementation details are described in sufficient detail in the supplementary methods of relevant papers^9,10^ to support replication with nonproprietary libraries. The AI system is not currently available for external use but interested parties should contact the corresponding authors of the relevant paper^10^ to be notified about external availability.
