# Supplementary material for: Impact of using artificial intelligence as a second reader in breast screening including arbitration
Source: Nat Cancer. 2026 Mar 10;7(3):507–21. doi: 10.1038/s43018-026-01128-z (PMC13035470; doi:10.1038/s43018-026-01128-z)
Supplement: Supplementary file 1 — Protocol. [file 43018_2026_1128_MOESM1_ESM.pdf]

nature cancer

Supplementary information https://doi.org/10.1038/s43018-026-01128-z

Impact of using artificial intelligence as a second reader in breast screening including arbitration

In the format provided by the authors and unedited

DocuSign Envelope ID: 8C37849F-C375-42F4-8310-CC1C11A57E2D

Imperial College London

Artificial Intelligence in Mammography Study (AIMS)

Clinical validation of an artificial intelligence system to improve the quality, efficiency and experience of breast cancer screening

Protocol v4.0, 21 August 2023

Main sponsor: Imperial College London

Funders: NIHR Artificial Intelligence (AI Award 2020 Phase 3) Competition

IRAS Project ID: 303782

REC reference: 22/EM/0038

Imperial College London

Contents

1 Study information **7**
1.1 Key study contacts 7
1.2 Investigators 7
1.3 Funding and support in kind 8
1.4 Role of trial sponsor and funder 8
1.5 Protocol contributors 8
1.6 Patient engagement in research programme 9
1.7 Study steering committee 9
2 Introduction 10
2.1 Background 10
2.2 Rationale for current study 11
3 Study objectives 12
3.1 Primary objective 12
3.2 Secondary objectives 12
4 Study design 12
5 Part A: Retrospective diagnostic accuracy study 13
5.1 Study aim 13
5.2 Dataset curation and study setting 13
5.3 Ground truth determination 14
5.4 Study endpoints 14
5.5 Participant entry 14
5.5.1 Inclusion criteria 14
5.5.2 Exclusion criteria 15
5.5.3 AI-system eligibility criteria 15
5.6 Methods and statistical analysis 15
5.7 Consent 16
5.8 Presentation of results 16
5.9 Identification of biases 16
6 Part B - Simulated usage of the AI system by readers in arbitration panels using retrospective data 17
6.1 Study aim 17
6.2 Dataset curation 17
6.3 Study endpoints 18

DocuSign Envelope ID: 8C37849F-C375-42F4-8310-CC1C11A57E2D

6.4 Methods 18
6.5 Statistical analysis 20
6.6 Participant recruitment and identification 22
6.7 Consent 22
6.8 Presentation of results 23
7 Patient and public engagement workshops 24
7.1 Aim 24
7.2 Methods 24
8 Data management 25
8.1 Data flows 25
8.2 Retention of data 26
9 Timescales 27
10 Ethical and regulatory considerations 28
10.1 Research Ethics Committee review 28
10.2 Peer review 28
10.3 Public and patient involvement 29
10.4 Protocol compliance 29
10.5 Monitoring and audit 29
10.6 Indemnity 29
10.7 Sponsor 30
10.8 Funding 30
10.9 Financial and other competing interests 30
10.10 Amendments 30
10.11 Assessment and management of risk 31
10.12 Potential for unintended bias 31
11 Expected outcomes of the study, Patient and Public Engagement and Dissemination 32
12 References 33
13 Appendix 1 34

DocuSign Envelope ID: 8C37849F-C375-42F4-8310-CC1C11A57E2D

This protocol has regard for the HRA guidance and order of content

RESEARCH REFERENCE NUMBERS:

- Public Health England Research Advisory Committee Ref: BSPRAC_0093

TRIAL REGISTRY NUMBER AND DATE: ISRCTN60839016 6th June 2022

DocuSign Envelope ID: 8C37849F-C375-42F4-8310-CC1C11A57E2D

PROTOCOL VERSION NUMBER AND DATE

Version Number	Date	Comment
1.0	5 January 2021	Final protocol submitted to REC
2.0	6 June 2022	Amendment for additional human factors work
3.0	24th November 2022	Follow-up for negative cases changed to 24 months
4.0	21 August 2023	Update information about ISO certification and addition of team member

Imperial College London

Signature page

The undersigned confirm that the following protocol has been agreed and accepted and that the Chief Investigator agrees to conduct the trial in compliance with the approved protocol and will adhere to the principles outlined in the Medicines for Human Use (Clinical Trials) Regulations 2004 (SI 2004/1031), amended regulations (SI 2006/1928) and any subsequent amendments of the clinical trial regulations, GCP guidelines, the Sponsor's (and any other relevant) SOPs, and other regulatory requirements as amended.

I agree to ensure that the confidential information contained in this document will not be used for any other purpose other than the evaluation or conduct of the clinical investigation without the prior written consent of the Sponsor

I also confirm that I will make the findings of the trial publically available through publication or other dissemination tools without any unnecessary delay and that an honest accurate and transparent account of the trial will be given; and that any discrepancies and serious breaches of GCP from the trial as planned in this protocol will be explained.

For and on behalf of the Study Sponsor:

Signature: Date:

DocuSigned by:
Cheuk Fung Wong
E6ED866F416A4F8...
.... 30-Aug-2023 ....../....../......

Name (please print):

Cheuk Fung Wong
....

Position: Research Governance and Quality Assurance Manager ....

Chief Investigator:

Signature: Date:

DocuSigned by:
Ara Darzi
447CB46CAAB7464...
.... 30-Aug-2023 ....../....../......

Name: (please print):

Ara Darzi
....

DocuSign Envelope ID: 8C37849F-C375-42F4-8310-CC1C11A57E2D

1 Study information

1.1 Key study contacts

Chief Investigator	Prof Ara Darzi, Lead for Research Translation, Director Institute of Global Health Innovation, Imperial College London, a.darzi@imperial.ac.uk
Sponsor	Institute of Global Health Innovation, Imperial College London
Funder(s)	NIHR Artificial Intelligence (AI Award 2020 Phase 3) Competition

1.2 Investigators

Name	Specify project role
Ara Darzi	Overall Chair and Lead for Imperial College London
Hutan Ashrafian	Lead for Research Translation
Deborah Cunningham	Co-lead for Imperial NHS Trust
Hema Purushothaman	Co-lead for Imperial NHS Trust
Mamatha Reddy	Co-lead for St George's NHS Trust
Lisanne Khoo	Co-lead for St George's NHS Trust
Lucy Warren	Lead for Royal Surrey NHS Foundation Trust
Fiona Gilbert	Co-Investigator and Scientific advisor
Anna Lawrence-Jones	PPI Lead, Imperial College
Shravya Shetty	Google Health engineering lead
Christopher Kelly	Google Health clinical lead
Jeremy Miles	Statistician, Google Health

Name(s) and address(es) of all medical and/or technical department(s) and/or institutions involved in the project, including any data processors and/or collaborators that will process the data:

- Imaging and Diagnostics Team, Google Health

- Department of Surgery, Imperial College London
- Breast Screening Service, Imperial College Healthcare NHS Trust
- Breast Screening Service, St George's University Hospital NHS Foundation Trust
- Department of Medical Physics, Royal Surrey NHS Foundation Trust
- Department of Radiology, Cambridge University

1.3 Funding and support in kind

FUNDER(S)	FINANCIAL AND NON FINANCIAL SUPPORT GIVEN
NIHR Artificial Intelligence (AI Award 2020 Phase 3) Competition	Financial
Google Health	Google staff costs across engineering, research scientists, user experience researchers, information governance team, security and privacy team<br>Technical infrastructure costs

1.4 Role of trial sponsor and funder

Imperial College London will act as the main Sponsor for this study. Delegated responsibilities will be assigned to the NHS trusts taking part in this study. The study Sponsor assumes overall responsibility for the initiation, management and funding of this study. The Sponsor is responsible for the design of this study, in addition to its conduct, data analysis and interpretation, and subsequent manuscript writing and dissemination of study findings. The Sponsor controls the final decision regarding any of these aspects of the study.

Funding - NIHR Artificial Intelligence (AI Award 2020 Phase 3) Competition. Google will not receive grant funding from the AI Award, and will cover the costs of its staff, technology, and infrastructure.

1.5 Protocol contributors

A wide range of disciplines have contributed to protocol development, including breast radiologists, clinical researchers, user experience/human factors researchers, medical physicists, engineering, statisticians, and patient engagement experts:

- Imperial College London: Ara Darzi, Hutan Ashrafian, Rachita Mallya, Anna Lawrence-Jones
- Imperial College Healthcare NHS Trust: Deborah Cunningham and Hema Purushothaman
- St George's University Hospitals NHS Foundation Trust: Mamatha Reddy, Lisanne Khoo

Imperial College London

- Royal Surrey NHS Foundation Trust: Mark Halling-Brown, Lucy Warren, Kenneth C. Young
- Cambridge University: Fiona Gilbert
- Google Health: Christopher Kelly, Lee Kupferman, Scott McKinney, Megumi Morigami, Rory Sayres, Amir Kiani, Shravya Shetty, Yetunde Ibitoye, Sam Fishman, Richard Sidebottom, Marcin Sieniek, Reena Chopra, Jeremy Miles

1.6 Patient engagement in research programme

Public representatives have been involved in all our original research. Two lay partners with experience of breast cancer participated in every stage of the initial model development, from training data specification and use-case, to evaluation strategy, iteration of our experiments, and preparing our publications.

We have recently recruited two additional lay partners to the steering committee to expand diversity of input. Lay partners are involved in decision making, design, and dissemination, and have been involved in the development of this research protocol. We have begun a series of workshops with wider groups of people that attend mammography screening or have been affected by cancer to discuss ideas, concerns, and expectations for the project.

1.7 Study steering committee

A study steering committee has been formed consisting of representatives from each organisation, alongside public representatives. This committee will meet at least monthly to ensure that the study is progressing well, review progress, and prepare relevant reports for the Sponsor.

2 Introduction

This project aims to evaluate the potential for artificial intelligence (AI)-enabled NHS breast screening to increase accuracy, safety, cost-effectiveness, and clinician/patient experience, while demonstrating evidence of clinical feasibility.

2.1 Background

1 in 8 women will be diagnosed with breast cancer in their lifetime. Breast screening aims to find cancers early, where treatment is more successful. In the UK, two readers assess each mammogram (x-ray of the breast), with disagreements reviewed by a panel. However, a radiologist workforce crisis threatens our screening programme's long term sustainability.

The UK is facing a 44% shortage in radiologists by 2025 (The Royal College of Radiologists 2021), while it is estimated that only 18% of screening hubs have sufficient staff to cope with current double reading requirements on a 3-yearly cycle (Public Health England 2016). If invitations were to be extended to women aged 47 to 73, as being investigated by the AgeX trial, the demands on the service would increase further (Moser et al. 2011). The negative impact of COVID-19 on breast screening in 2020 only adds to the increased burden expected over the coming years as services catch up. Breast Cancer Now estimated that almost 1 million women have missed their breast screen during the pandemic (Breast Cancer Now 2020), and Macmillan's analysis of the rate of recovery suggests it would take 20 months to work through the current backlog if activity was increased to 10% above pre-pandemic levels (Macmillan Cancer Support 2020). In addition, there are calls for the UK to adopt two-yearly screening, mirroring most other European countries, which would be unfeasible given current workforce pressures, without compromising quality. We believe AI-enabled screening can play an important role in future-proofing the UK's Breast Screening Programme.

Google's Mammo-Reader is an AI-powered independent mammography reader product for double-read breast cancer screening workflows. It analyses two-dimensional full field digital mammography to give a normal/abnormal screening determination, and highlights suspicious regions of interest. In a study published in Nature (McKinney et al. 2020), the AI system was able to demonstrate performance close to that of double reading with arbitration (statistically non-inferior) and superior to the first reader. We believe that deploying this technology as a second reader has the potential to: (1) ultimately improve patient outcomes through improved accuracy and reduced variability; (2) modify the reader / radiologist workload mitigating the current workforce challenges and even allow expansion to alternative screening strategies such as biannual or personalised stratified approaches; and (3) reduce time to results, improving patient experience. In addition, the discrepancy between a radiologist and the AI system is greater than two humans, suggesting that it may be possible to detect a greater number of cancers (higher overall sensitivity), albeit at a cost of greater number of cases for arbitration.

The AI system is intended to be deployed as a second reader within the UK breast screening programme, integrated securely into the National Breast Screening System (NBSS) and clinical imaging PACS. In our primary proposed workflow, a screen reading expert will read all cases first. Once the first reader has submitted their assessment, and eligibility checks have been completed the AI system's assessment will be made available. The AI system's decision will be compared to

Imperial College London

the first reader. In the estimated 10% disagreements, these cases will be referred to an arbitration panel for final determination (following existing local site arbitration rules). We believe this workflow promotes safety by ensuring all AI system decisions require independent agreement from human readers to influence a woman's care, and that all disagreements are reviewed by expert arbitration panels.

This mammography product builds upon Google/DeepMind's world-leading expertise in pioneering artificial intelligence technologies, unique experience in scaling multiple products and services to billions of users around the world, and world-leading Cloud infrastructure with significant experience in healthcare applications. Complementing Google's technology platform, Google Health's cross-functional regulatory, information governance, quality and clinical infrastructure provides a unique environment to build safe, secure, robust, reliable, fair, and accurate clinically validated products to achieve patient impact at scale.

The AI system has been developed under a Quality Management System adhering to CE certification requirements, and is currently both ISO 13485 and ISO 27001 compliant.
The certifications will expire in early 2024 after the anticipated study completion date.

2.2 Rationale for current study

This project includes two main components. Firstly, we will compare the AI system to radiologists in large historical populations at two NHS sites with diverse multicultural patient populations. This will allow detailed assessments of accuracy; ensure fair, equitable performance; and enable modelling of workforce and economic impacts. This will complement other evaluation studies being performed by Google.

Secondly, we will perform a large diagnostic study to re-read historical mammograms from approximately 50,000 women across two hospitals to explore how their expert radiologists in arbitration panels interact with the AI system when used in place of the second radiologist. This will explore the complex human factors involved, measure overall system accuracy of AI-enabled screening, and enable assessment of NHS health economic impacts.

Interventional use of the AI system within the health system cannot be commenced before comprehensive modelling of likely clinical, workflow, and economic impacts. This proposal outlines our plans to translate this ground-breaking research towards real world patient impact through retrospective diagnostic accuracy studies and large scale consensus panel reader studies. The next phase of this work will include prospective observational feasibility studies to test the AI system running 'silently' within each hospital. Through this work, we aim to provide the necessary evidence to support progression to future interventional use in a way that delivers measurable benefits to public health.

Imperial College London

3 Study objectives

Based upon our recent published work (McKinney et al. 2020) and subsequent progress towards development of a regulated medical device, we hypothesise that:

- Our novel AI system for breast cancer screening demonstrates the appropriate accuracy, safety, acceptability, and cost effectiveness required for use as an independent reader within the NHS breast screening programme

3.1 Primary objective

a) To test accuracy of the system acting as an independent second reader in large retrospective NHS breast screening datasets, compared against historical clinical reader accuracy, using cases from multiple NHS screening sites and mammography devices, assessed against a robust ground truth of biopsy-proven cancer over 39 months.

3.2 Secondary objectives

a) Assess the performance and human factors of a simulated specialist arbitration panel when the AI system is used as the second reader
b) Perform detailed analyses to identify underperformance in clinical or demographic subgroups, and any unintended biases. Understand the types of cancers detected by AI to allow an assessment of potential changes to the balance of benefits and harms including potential overdiagnosis.
c) Perform detailed clinical, workflow, productivity and economic analyses to assess overall benefit to the screening service
d) Understand patient and public ideas, concerns and expectations of AI-enabled breast cancer screening through workshops and surveys

4 Study design

The study consists of two components: Part A: retrospective diagnostic accuracy study, and Part B: simulated usage of the AI system by readers in arbitration panels using retrospective data.

The anticipated study start date is 1st January 2022. The duration of the study is 24 months.

5 Part A: Retrospective diagnostic accuracy study

5.1 Study aim

The retrospective diagnostic accuracy study aims to evaluate accuracy, generalisation and fairness of the AI system, compared to contemporaneous clinical decisions, using large historical pseudonymised screening datasets from at least two UK screening sites with a range of clinical practice, collected previously during routine screening, with subsequent longitudinal cancer follow up. This study will allow a precise evaluation of AI system performance, bias, and safety using datasets where long-term cancer outcomes are available for accurate ground truth determination.

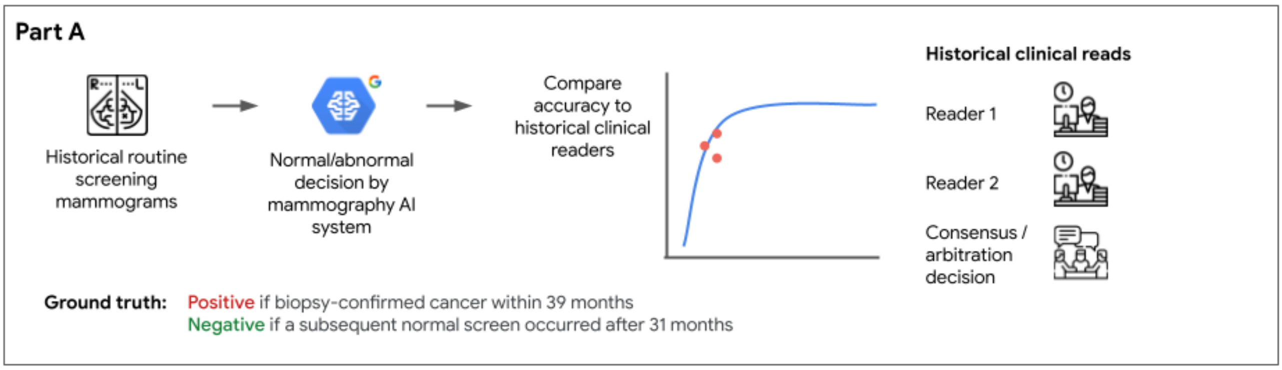

5.2 Dataset curation and study setting

A test dataset from each screening site will include at least 25,000 women per site aged between 50 and 70 who have previously attended for routine screening from January 2016 onwards. Cases will be selected randomly in order to curate a dataset that is representative of the screening population. Datasets will include all screening mammograms relevant to the selected screening visit, including technical recalls comprising mediolateral oblique and craniocaudal views of the left and right breasts, all reader opinions including the arbitration result (if applicable), and metadata associated with follow-up treatment. These data have been collected in the course of normal clinical care, and no primary data will be collected. No data from women included in datasets used for AI system development will be included in these test datasets.

Importantly, imaging and clinical data will be collected under existing ethical permissions for the OPTIMAM database (REC references 14/SC/0258 and 19/SC/0284), using existing infrastructure and technical methodology. No additional permissions are being sought for Part A. Data will be shared with Google Health under existing ethical approvals, existing OPTIMAM steering group approvals, and an existing data sharing agreement.

The OPTIMAM Mammography Image Database (Halling-Brown et al. 2021) is a large curated and centralised database of mammogram images and associated clinical data from multiple NHS Breast Cancer screening sites. The database contains unprocessed and processed medical images, associated expert-determined region of interest annotations and clinical data relating to screen detected and interval cancers. The process of image collection, annotation and storage is almost fully automated and is very adaptable, allowing for quick and easy expansion to disparate imaging sites. The ongoing collection of data is funded by Cancer Research UK and the database

is hosted and managed by Royal Surrey NHS Foundation Trust. The resource has been designed to be shared for research purposes. However, the consortium will update the OPTIMAM database steering committee of the research being undertaken.

Data will be collected from two NHS screening services at St George's University Hospitals NHS Foundation Trust and Imperial College Healthcare NHS Trust. We may enrol additional sites that are part of the OPTIMAM database if required to achieve acceptable confidence intervals and diversity of representation for the study's secondary endpoints.

Google Health will not receive identifiable patient information, and all dataset collection and linkage will take place on hospital premises (see Section 8. Data management).

5.3 Ground truth determination

Cases will be considered positive if they received a biopsy-confirmed diagnosis of cancer within 39 months following the screening visit. Negative cases will require a negative result from the study screening visit, and another negative result at the subsequent screening visit at least 24 months later.

5.4 Study endpoints

Primary endpoints for the study will be:

- AI system cancer detection sensitivity and specificity (diagnostic accuracy matrix) compared to first, second and consensus reader decisions, measured against ground truth definition above.

Secondary endpoints will include:

- Subgroup performance by factors including cancer type and grade, primary tumour size, patient age, breast density, prior cancer, prevalent and incident screens, ethnicity, device manufacturer, socioeconomic status, and screening site.
- AUC-ROC for cancer detection, positive predictive value, negative predictive value, cancer detection rate, case recall rate.
- System performance in confirmed interval cancers (percentage of historical interval cancers that the AI system flagged for recall, and qualitative agreement of the localisation in the original screening mammogram with the presence/absence of true radiological evidence).
- AI system localisation performance (if lesion position data available).
- Analysis of failure cases.
- Percentage of women that meet the eligibility criteria.
- Simulations of workforce impact assessment and health economic modelling.

5.5 Participant entry

5.5.1 Inclusion criteria

- Women undergoing routine breast cancer screening (age 50-70) as part of the national breast screening programme from January 2016 onwards.

- Mammography images acquired using Hologic/Lorad, Siemens, or GE devices.

5.5.2 Exclusion criteria

- None

5.5.3 AI-system eligibility criteria

In the first instance, the AI system is intended for use in routine screening patients that make up the bulk of the screening programme workload. The following criteria apply for a case to be deemed eligible to be read by the AI. The number of cases deemed ineligible will be recorded.

<u>Eligible</u>

- Women undergoing routine breast cancer screening (age 50-70), as part of the national breast screening programme.
- Mammography images acquired using Hologic/Lorad, Siemens, or GE devices.

<u>Ineligible</u>

- Women attending an assessment clinic or symptomatic clinic (i.e. not routine screening).
- Women undergoing annual screening due to:
    - High risk (lifetime risk >30% - e.g. faulty BRCA1, BRCA2, TP53)
    - Moderate risk (lifetime risk 17-30%)
    - Personal stratified follow up (e.g. indeterminate B3 lesions)
- Presence of breast implants.
- Screens with incomplete (<4 standard screening views - e.g. due to abandoned screen)
- Poor diagnostic quality imaging (which would be repeated).
- Non-standard acquisitions beyond the routine 4 screening views.
- For negative or benign cases, women without a negative follow up screen approximately 3 years later (at least 24 months after initial screen), as this would preclude determination of a robust ground truth.

These eligibility checks will be identified by checking DICOM labels (e.g. for implants, views and number of images available) and NBSS reports for technical repeats and recalls, annual screens, and diagnosis and follow-up screen results. Eligibility checks will be applied in future prospective feasibility testing of the AI system within an NHS workflow in a similar manner. In our future work, we plan to address these excluded cohorts to increase the eligible population available to the AI system.

5.6 Methods and statistical analysis

We plan to replicate the methodology used previously at two NHS screening sites with diverse patient populations (McKinney et al. 2020).

Cancer will be defined as biopsy-confirmed cancer within 39 months of index mammogram, while healthy cases will require an additional normal mammogram at the next screen. The study's primary endpoint will be powered at a site level using a cancer sensitivity non-inferiority margin of 5% and target power of at least 80%. This will require approximately 50,000 women overall that meet the eligibility criteria (25,000 per site), with a population prevalence of 400 cancer cases

overall (200 per site). We may enrol additional OPTIMAM sites or collect additional data from existing sites if required to achieve acceptable confidence intervals for our secondary endpoints.

For each site, we will compare the sensitivity and specificity of the readers with that of a pre-specified thresholded score from the AI system. The pre-specified threshold will be based on the performance of the AI system on a separate retrospective dataset that does not overlap with the cases included in this study, and will be optimised to mirror a radiologist in terms of balanced sensitivity and specificity. Confidence intervals on the difference will use Wald intervals (Fagerland, Lydersen, and Laake 2014), and a Wald test will be used for non-inferiority (Liu et al. 2002). Both will use the Obuchowski variance estimate (Obuchowski 1998). For non-inferiority comparisons, we plan to use a 5% absolute margin (as used in our Nature paper, and generally more stringent than the literature). Superiority comparisons on the UK data will be conducted using Obuchowski's extension of the two-sided McNemar test for clustered data. We will use a statistical significance threshold of 0.05, and will correct for multiple comparisons using the Holm–Bonferroni method.

To evaluate the stand-alone performance of the AI system, the area under the receiver operating characteristic (AUC-ROC) curves will be estimated using the normalised Wilcoxon (Mann–Whitney) U statistic (Mann and Whitney 1947). Non-parametric confidence intervals on the AUC-ROC will be computed with DeLong's method (DeLong, DeLong, and Clarke-Pearson 1988).

We will perform an additional analysis using a relaxed ground truth, where a follow up negative screen is not required. This is because women who are attending for their final screen around the age of 70 will not have a confirmatory negative screen. However, non-screen-detected cancers detected within a 3 year window following the screen will be available through NBSS, and so positive cases will still be appropriately recorded.

5.7 Consent

This study will only involve secondary analysis of anonymised datasets curated as part of the OPTIMAM database (REC references 14/SC/0258 and 19/SC/0284). Informed consent is not sought because we do not obtain or store identifiable data. The only processing of patient identifiable data is the de-identification process itself which takes place at the point of collection on a dedicated research server, in an automated manner, performed by the OPTIMAM project under their existing approved processes.

5.8 Presentation of results

Results will be presented graphically through ROC plots and confusion matrices, alongside standard sensitivity, specificity, positive/negative predictive value, cancer detection rate and recall rate measures.

5.9 Identification of biases

To avoid unintended future harm from the AI system, we will carefully analyse the data to identify the presence of any hidden stratifiers in the data that may lead to suboptimal performance in certain patient/disease groups.

6 Part B - Simulated usage of the AI system by readers in arbitration panels using retrospective data

6.1 Study aim

This study of retrospective breast cancer screening mammography aims to understand the impact of introducing an AI system as a second reader into a double reader workflow, through a large-scale end-to-end study of arbitration performed at two diverse NHS sites. Each site will re-assess their own historical arbitration cases in a simulated setting, using their own mammography readers, and their own arbitration clinical pathways as part of the study.

Study aims include:

- To assess end-to-end clinical efficacy of the double-reading screening system using AI as one of the two readers at two hospitals through large-scale simulated arbitration panels.
- To understand if and how UK readers will change their recall behaviour when AI is incorporated into the screening pathway
- To understand the impact on interval cancers of incorporating AI into the pathway
- To understand whether clinical pathway variability in current practice has an impact on cancer detection with AI through inclusion of two screening centres with different clinical workflows
- To understand human factors implications, including identification of potential areas of bias, and develop appropriate mitigations.
- Quantify workflow impacts and perform a full health economic assessment.

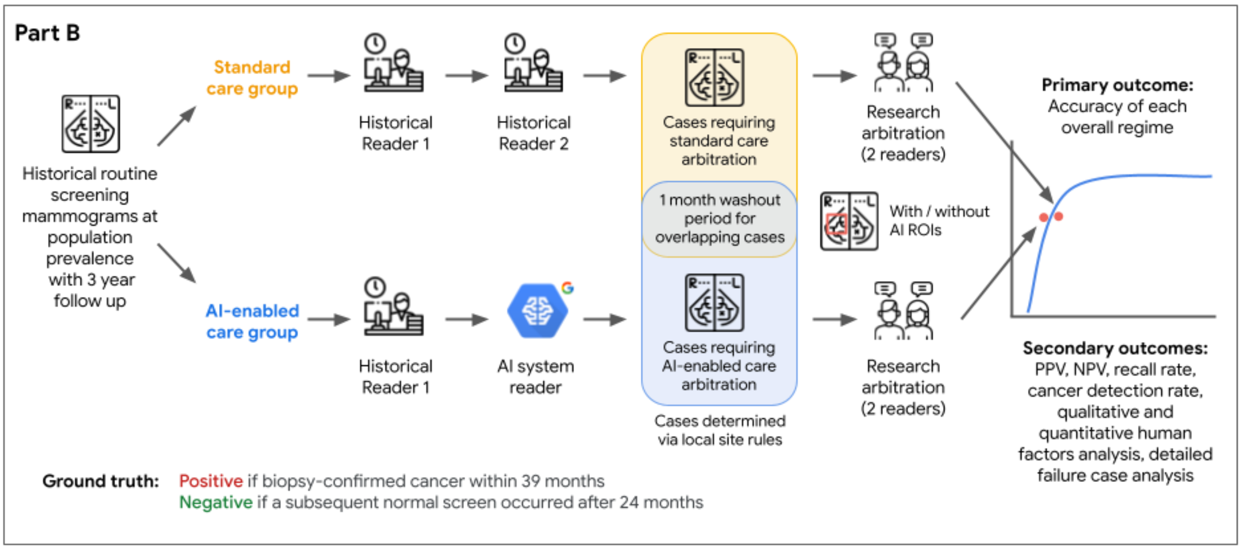

6.2 Dataset curation

Imperial College London

Cases in the Part A dataset that previously required arbitration, or that would require arbitration where AI was used as the second reader will be included in the Part B study. Each site will use its own historical data and clinical readers for the reader study.

In order to ensure a realistic arbitration reading setting for the Part B study, we will need to retrieve and transcribe the paper notes that historical readers used to record their opinion onto new study-specific sheets that have been de-identified with a study number only, and only include the required information for each arm of the study. This will ensure that the arbitration sheets are anonymised to the Readers and researchers involved in the study.

NHS Trust-employed research radiographers (part of the care team) will perform the curation of the study arbitration sheets for each case. Patients in the part B study will undergo re-identification by the local site using the pseudonym lookup table accessed by the Trust's research radiographer through the internal web portal of the research server. T
h radiographers account, the web portal will automatically output a pseudonym lookup (NHS numbers and pseudonyms). The research radiographer will then use the NHS numbers (hospital numbers may be required in some circumstances) to access paper and electronic records, for the purpose of transcribing historical clinical notes from prior screening visits into a de-identified format. See Section 8 "Data management" for further details on data processing.

For the Reader study itself, film readers will only access anonymised data.

6.3 Study endpoints

- **Primary endpoints:** Sensitivity and specificity of cancer detection using a 39 month follow up window, compared between the standard care group and the AI-enabled care group.
- **Secondary endpoints including:** Positive predictive value, negative predictive value, cancer detection rate, case recall rate, number of arbitrations in both arms, average time taken to arbitrate a single case, accuracy analysis by all relevant subgroups mentioned in Part A, health economic modelling based upon study findings, difference between study arbitration decisions and historical arbitration decisions, differences in accuracy measures between readers with different levels of experience.

6.4 Methods

- **Dataset:** Same dataset as defined in Part A, with the same inclusion and exclusion criteria.
- **Reader participants:** Radiologist and radiographer film readers will be recruited at each site and grouped into local arbitration pairs. From initial investigations, we anticipate approximately 10 readers grouped into 5 pairs per site will be feasible, performing one research arbitration session of up to 2 hours per week. Each reader will be consented prior to participation. Readers will undertake research sessions outside of their normal working hours so clinical throughput is not impacted. Reading sessions will be paid at the readers' usual rate.
- **Two arbitration methods (standard / AI-enabled):** Cases will be determined for arbitration for both AI-enabled and standard care using local site protocol - for example, St

DocuSign Envelope ID: 8C37849F-C375-42F4-8310-CC1C11A57E2D

George's arbitrates all cancer recalls, Imperial arbitrates only discordant recalls. The standard care group is essential to control for the possibility that readers will perform differently in a research setting vs their usual clinical setting.

  - **"AI-enabled care group":** Historical Reader 1 with AI system as Reader 2
  - **"Control standard care group":** Historical clinical Reader 1 and Reader 2

- **Reader study summary:** Readers will be onboarded including practice introductory cases, training to understand the AI system (features, benefits, limitations) and the RiViewer software used for reviewing the cases (see Appendix 1 for more details). Arbitration panels of two readers each will read groups of AI-enabled care cases and standard care cases as they would in clinical practice. Each case will be read with mammography images accompanied by anonymised prior mammograms (if available), historical notes from both original clinical readers (for standard group), or original human reader 1 plus a graphical display of suspicious regions of interest from the AI system (for AI-enabled group). The readers will be asked to complete an electronic Case Report Form for each case, including recall decision, confidence score, highlighted suspicious regions of interest if applicable, along with general comments about the case. For cases included in both groups, cases will be read in a randomised order between arms, with a washout period of at least 1 month between reads.
- **AI viewer:** The AI output will be displayed as a DICOM image on a separate monitor. The output displays the breast-level assessment (either normal or abnormal), and will be the same for both views of the same breast. Regions of interest (ROIs) will be overlaid on the mammogram images, with additional text providing information on the number of ROIs and on which image they have been detected.
- **Human factors research:**
  - Analyses of case report form data, including comments and confidence scores, provided during each arbitration panel decision.
  - Explore additional human factors with readers before, during, and after participating in the study. This may include reviewing additional cases, submitting surveys and/or participating in semi-structured interviews/focus groups about perception of AI vs human reader, such as:
    - Perceived accuracy of the AI system
    - Perceived task load of arbitration with the AI compared to without
    - Perceived self-efficacy for arbitration readers in each arm

    Interviews will be conducted via video call between readers and the research team, and may be audio or video recorded for the purposes of transcription into an anonymised format. Recordings will be deleted after transcription.
- **Post-hoc detailed failure analysis:**
  - Review of failure cases, including using subsequent positive mammograms to identify regions of interest in false negative mammograms.
  - Review of all interval cancers and associated thematic analyses.

- Quantitative review of arbitration readers' bias towards incorrect AI decisions.

- **Health economic evaluation:**
    - In collaboration with health economist specialists at Google Health and Institute of Global Health Innovation, perform detailed health economic modelling based upon results from Part A and B of this project, in order to provide information to the national screening programme to help evaluate AI technologies.

6.5 Statistical analysis

The arbitration reader study has two arms: traditional workflow (historical R1 + historical R2 → study arbitration) and an AI-enabled workflow (historical R1 + study AI R2 → study arbitration). Each case is evaluated according to both regimes. The study will compare the final screening decisions reached for all cases, including the research arbitration panel if applicable, between the two study arms.

The study will be powered for sensitivity using a 5% non-inferiority margin, with at least 80% power. The same dataset as used in part A of approximately 50,000 women (25,000 from both Imperial College Healthcare NHS Trust and St George's University Hospitals NHS Foundation Trust) that meet the AI eligibility criteria will be included in the study, resulting in an estimated 4,000–6,000 arbitration cases per site, varying depending on local arbitration rules.

Difference between the two arms will be analysed using a matched-pair comparison via a Wald test (Liu et al. 2002). We aim to achieve non-inferiority on sensitivity of cancer detection, so the power calculation is based upon the number of positive cases required. Given the low prevalence of cancer at screening, any specificity comparisons will be abundantly powered.

To perform the power calculation, we first generate R1, R2, and AI model decisions according to the joint distribution observed from a similar OPTIMAM dataset previously used for model validation:

R1	R2	M	*p*
0	0	0	0.23
0	0	1	0.085
0	1	0	0.026
0	1	1	0.046
1	0	0	0.017
1	0	1	0.014
1	1	0	0.089
1	1	1	0.49

Key: 1 = recall, 0 = no recall. n = 1250. p = observed joint distribution in the dataset (# of positive cases where R1=x, R2=y, M=z) / (# of positive cases total)

Imperial College London

Next we generate arbitration reads for each arm based on the marginal recall rate observed among arbitrated positives and an agreement parameter *k*. Concretely, this is the rate of agreement between the arbitration reads in the two arms, assuming they were both to arbitrate a given case. We've evaluated values of 0.7, 0.8 and 0.9. We note that the agreement rate between model and arbitration reader on discordant positives is already 68%, so this is likely to set a floor on the actual value of *k*. Our power calculations are therefore based upon a likely lowest agreement value of 70%.

There are two assumptions of this simulation:

1. Cases are arbitrated if:
   a. For Imperial:
      i. Control: R1 ≠ R2
      ii. Experiment: R1 ≠ model
   b. For St George's:
      i. Control: R1 and/or R2 recalls the case
      ii. Experiment: R1 and/or model recalls the case
2. The arbitration decision is independent of *which* of R1 or R2 (or the model) asked for recall.

Imperial College Healthcare NHS Trust: only discordant cases are arbitrated

Simulation results where only discordant cases are arbitrated are as follows:

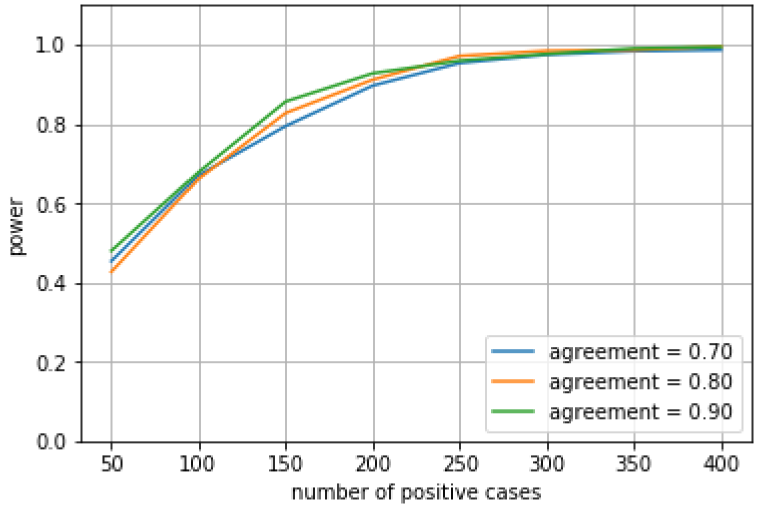

Under this arbitration regime, our modelling suggests that the power is relatively robust to the agreement parameter.

Therefore for any reasonable agreement value above 70%, the study is expected to achieve at least 90% power with minimum 200 cancer positive cases from Imperial.

St George's University Hospitals NHS Foundation Trust: all recalls are arbitrated

Simulation results where all cancer recalls are arbitrated are as follows:

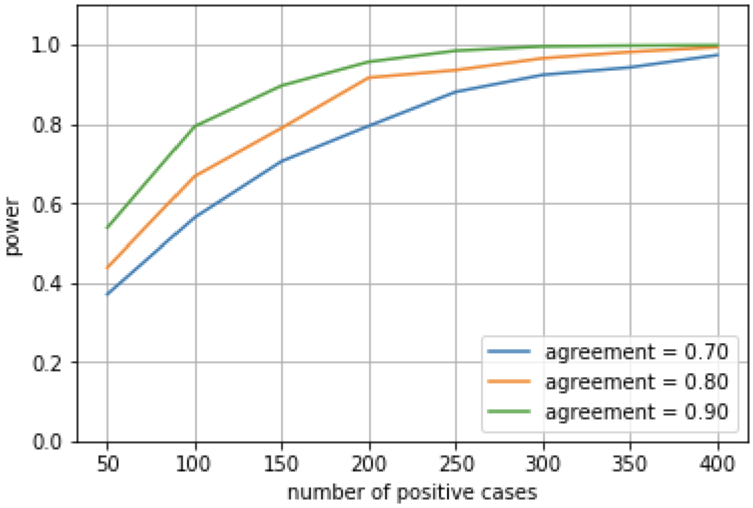

In this setting where all recalls are arbitrated, the power appears more dependent on the rate of agreement between the arbitration reads in the two arms, assuming both arms arbitrate a given case. We do not yet have pilot data to reliably estimate this agreement rate, and there are no previous studies upon which we can base our assumptions. Assuming an agreement of 80%, the study is expected to achieve 90% power with 200 cancer positive cases from St George's. If the agreement between arms was 70% (our previously stated expected minimum), then 200 cancer positive cases would still give the study at this site 80% power.

6.6 Participant recruitment and identification

Screening participants: All data used for this study will be historical and collected in the course of routine clinical care. The OPTIMAM team at Royal Surrey NHS Foundation Trust will select cases and collect data for this study through the OPTIMAM project. Participants will be selected from the available routine screening population who meet eligibility criteria in order to achieve target sample sizes and to be representative of the screened population. Publicity including posters at screening sites will inform patients of the OPTIMAM project (through which the data will be collected).

Film reading readers: Participants in the reader study will be voluntarily recruited from both clinical sites. Readers must be either breast screening radiologists or film reading radiographers that participate in arbitration. There is no other exclusion criteria. The sites involved in this study will be funded for the readers' time through the AI Award. There will be no direct incentives provided to the readers. Readers can withdraw at any time without giving a reason, but any information about the readers or any research data that has already been provided will be kept and used for analysis.

6.7 Consent

Screening participants: Informed consent is not sought because we do not obtain or store identifiable data. This study will involve secondary analysis of anonymised imaging datasets

curated as part of the OPTIMAM database (REC references 14/SC/0258 and 19/SC/0284). OPTIMAM's de-identification process takes place at the point of collection on a dedicated research server, in an automated manner, performed by the OPTIMAM project under their existing approved processes. Posters and information are clearly available at screening sites to notify women of the sites' involvement in this project. In addition to the OPTIMAM dataset, Trust research radiographers will curate de-identified arbitration working sheets from historical records. Once curated, these de-identified sheets would be considered anonymised to the Readers and research team, as they do not have access to the pseudonym keys required.

Film reading readers: Readers will be asked to consent before involvement in the reader study. Participant information will be provided with a concise summary of their involvement in the study, including familiarisation with this protocol. Informed consent forms will be completed before beginning the study.

6.8 Presentation of results

On completion of the study, the data will be analysed and tabulated, and a final study report will be prepared. The study report will be published in a peer-reviewed academic journal with open access as soon as possible following the report finalisation.

Imperial College London

7 Patient and public engagement workshops

7.1 Aim

- To understand public/patient ideas, concerns and expectations about the use of AI in breast screening mammography, in parallel with Parts A and B.

7.2 Methods

We successfully received Imperial College London Research Ethical Committee Approval on 17/02/2021 to conduct patient and public involvement and engagement workshops for our study titled 'Assessment of patient and public acceptability of an artificial intelligence (AI) based mammographic screening tool to improve quality, efficiency and experience of breast cancer screening' (ICREC reference: 21IC6635). We have started and will continue to run workshops with diverse groups of up to 14 participants who have had experience of breast cancer or have previously experienced routine mammography screening. These workshops are co-facilitated by a lay partner. We will discuss patients' ideas, concerns and expectations for the project, which will feed into project design. All lay partners will be appropriately trained and have access needs supported. Due to COVID-19, we may have to carry out meetings remotely. To ensure our opportunities are inclusive, we will add a phone number to the workshop advert and advertise through community groups. We will send guidance and pay for any dongles for those without internet access. Members will be paid £25/hour and £5 for any online interaction. We will evaluate the impact of our PPI using the GRIPP2 academic tool and asking for feedback.

Imperial College London

8 Data management

Data management for OPTIMAM which supplies data required for the retrospective study (part A) and reader study (part B) are described in detail in the separate "Management protocol for the OPTIMAM Image Database" v2.6 (14/10/2019, REC reference 19/SC/0284). Briefly, this protocol provides a comprehensive outline of the purpose, operation, methods, policies and governance of the OPTIMAM Image Database. It describes the procedures used to collect and store mammographic images for research.

Data will be pseudonymised by the Royal Surrey NHS Foundation Trust team at the point of collection using DICOM 142 supplement compliance tools. This supplement is a standard for de-identification of data in DICOM files. Pseudonym lookup tables will be maintained on secure servers at the clinical collection sites and access will be restricted to staff involved in the patients' clinical care or data managers with specific approval to access the patient data for the purpose of pseudonymisation. The data managers will have NHS letters of access permitting the level of access required for the pseudonymisation process. All data shared with research staff will have already been de-identified and they will have no access to the pseudonym lookup tables. To avoid re-identification the patient data within the database is restricted to year of birth and sex. Attempts to identify subjects are forbidden by data access agreements. Access to the pseudonymisation lookup lists held securely at the clinical sites is limited to staff with responsibility for clinical care or data managers with specific approval from the clinical site.

8.1 Data flows

For both screening sites, Imperial College Healthcare NHS Foundation Trust and St George's University Hospitals NHS Foundation Trust, data from the NBSS database (1) and PACS (2) will be curated and pseudonymised (4) under existing ethical permissions for the OPTIMAM database (OMI-DB) (REC reference 19/SC/0284), using existing infrastructure and technical practices. This retrospective dataset will undergo pseudonymisation a second time by the OPTIMAM team, providing a unique set of anonymised identifiers for the purpose of this study (5). The dataset will then be transferred to and analysed by the Google AI system on secure cloud computing infrastructure (6, 7, 8).

DocuSign Envelope ID: 8C37849F-C375-42F4-8310-CC1C11A57E2D

Data flow diagram for both Part A and B

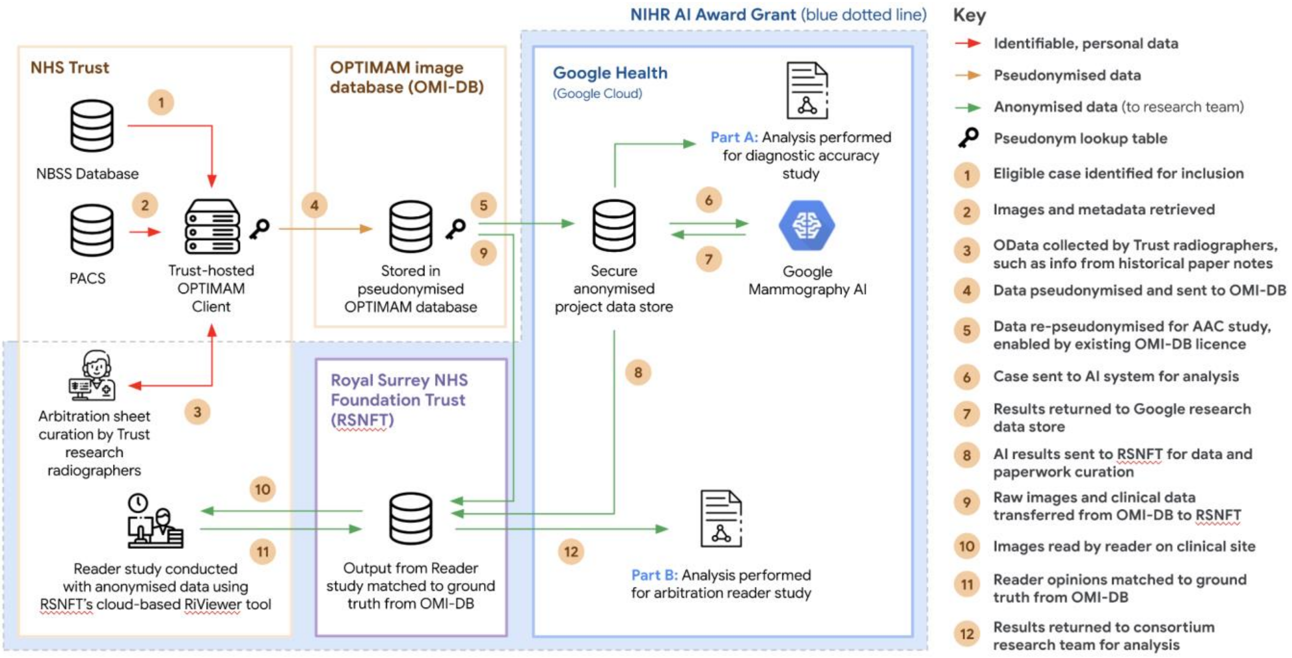

For Part B, an estimated 10% of cases will undergo arbitration in a reader study. For this work, these cases will be re-identified at each Trust using the pseudonym lookup table provided by OMI-DB to the local clinical sites. Only the local clinical sites will carry out the re-identification, and any identifiable data will only be accessible by the Trust research radiographer. The research radiographer will use the NHS Number (hospital number may be required in some circumstances) to access the paper or electronic screening records for these patients to transcribe historical clinical notes from prior screening visits onto a new anonymised format to ensure blinding to the original human read (3). The data will be pseudonymised again by the OPTIMAM team for the reader study, retaining the unique set of anonymised identifiers as for Part B (4, 5). The dataset will be transferred to secure Google Cloud storage, and onwards to Royal Surrey NHS Foundation Trust (8) to conduct the reader study at the local Trust sites using their cloud-based RiViewer software (see Appendix 1 for more details) (9). The output from the reader study will be matched to the ground truth from the OMI-DB (10), and returned to Google Cloud storage (11) for analysis of the collected data.

8.2 Retention of data

Anonymised research data generated by the study will be archived after study completion for a period of 10 years, as per Imperial College London policy. Data will be stored securely at the Institute of Global Health Innovation at Imperial College London. Access will be granted to the Principal Investigator and their deputies only, with security controlled through two-factor authentication. Google Health will retain anonymised study data for a maximum period of 10 years. The study dataset will be stored in dedicated, encrypted, secure health research storage, with two-factor authentication, and strict access control lists that are limited to researchers directly working on this research study.

DocuSign Envelope ID: 8C37849F-C375-42F4-8310-CC1C11A57E2D

9 Timescales

The study design and site set up commenced in 2021. The studies described in this protocol are planned for 2022/23. The planned timescale and milestones are described in the following chart:

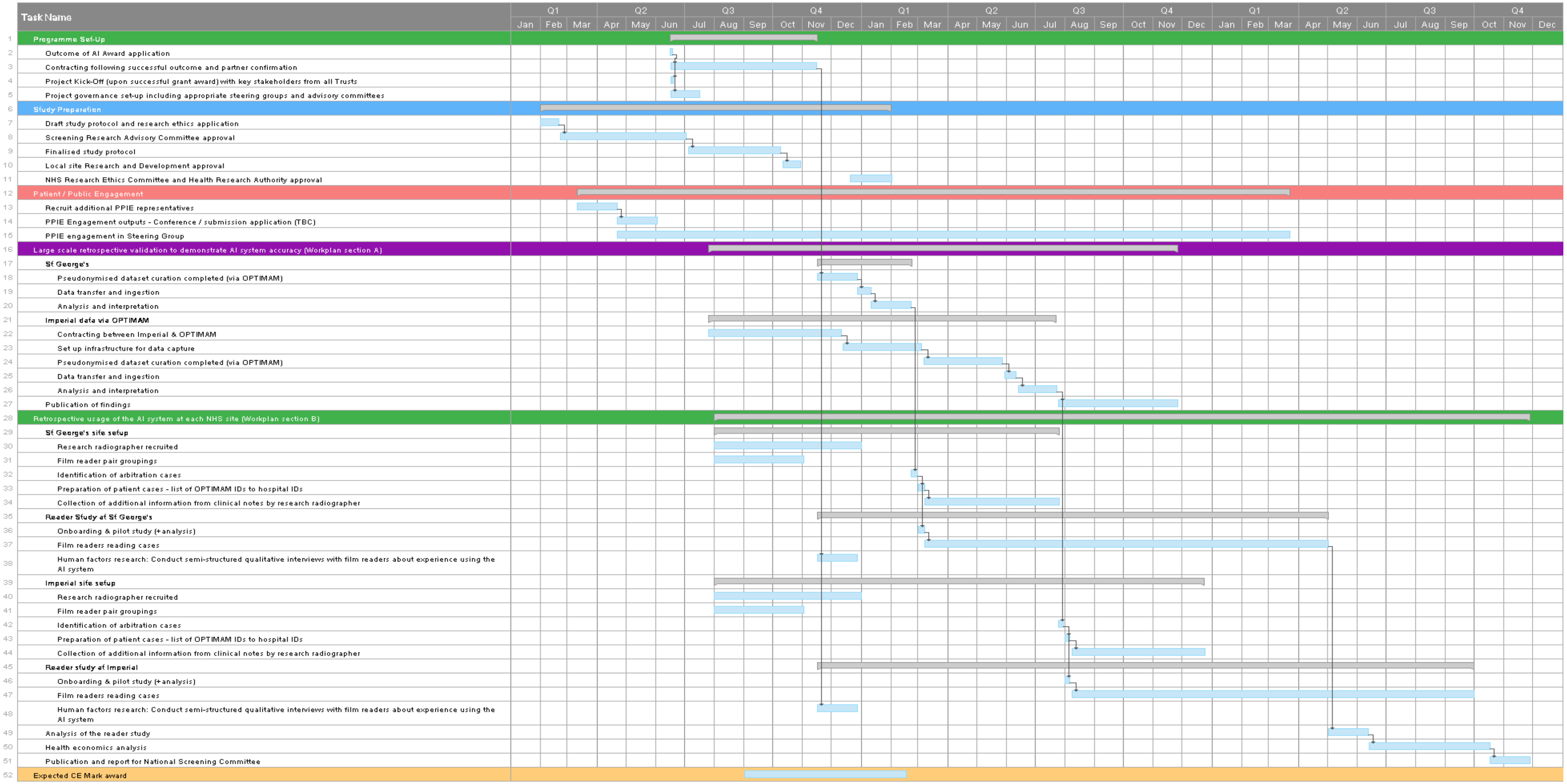

Imperial College London

10 Ethical and regulatory considerations

10.1 Research Ethics Committee review

- The Study Coordination Centre has obtained approval from the Nottingham Research Ethics Committee (REC) and Health Research Authority (HRA) for the trial protocol, and other relevant documents.
- The study must also receive confirmation of capacity and capability from each participating NHS Trust before accepting participants into the study or any research activity is carried out. The study will be conducted in accordance with the recommendations for physicians involved in research on human subjects adopted by the 18th World Medical Assembly, Helsinki 1964 and later revisions.
- Substantial amendments that require review by REC will not be implemented until the REC grants a favourable opinion for the trial.
- All correspondence with the REC will be retained in the Trial Master File/Investigator Site File.
- An annual progress report (APR) will be submitted to the REC within 30 days of the anniversary date on which the favourable opinion was given, and annually until the trial is declared ended
- It is the Chief Investigator's responsibility to produce the annual reports as required.
- The Chief Investigator will notify the REC of the end of the study
- If the study is ended prematurely, the Chief Investigator will notify the REC, including the reasons for the premature termination
- Within one year after the end of the trial, the Chief Investigator will submit a final report with the results, including any publications/abstracts, to the REC.

10.2 Peer review

This study is funded by the Artificial Intelligence in Health and Care Award. As part of the application process for this award, the study underwent independent external review by the Accelerated Access Collaborative in partnership with NHSX and the National Institute for Health Research (NIHR), in addition to the Breast Screening Research Advisory Committee (RAC). The protocol underwent further review by the NHS Accelerated Access Collaborative Evaluation Advisory Group (EAG) in December 2021. The study has been reviewed by the Imperial College London Research Governance & Integrity (RGIT) peer review service.

DocuSign Envelope ID: 8C37849F-C375-42F4-8310-CC1C11A57E2D

10.3 Public and patient involvement

From project inception in 2016, we have had 2 lay partners with personal experience of breast cancer as part of a committee comprising approximately 10 UK breast screening radiologists and academics. They were involved in every stage of the initial development, from training data specification and use-case, to evaluation strategy and iteration of experiments, and when writing the manuscript for our publication. This ensured that our AI system meets the needs of the screening programme, radiologists and patients alike - classifying occurrence of cancer within 3 years (designed around the NHS programme interval), acting as second reader (advised by Public Health England as their most impactful application), and validated in a large representative dataset.

We will meaningfully involve lay partners throughout this project. We have recruited 2 additional lay partners to join our steering committee to increase the diversity of voices and in case members cannot attend. Lay partners will be directly involved in decision making, design and dissemination of findings. In addition, they will provide input into infographics and blogs aimed at the general public.

All lay partners will be appropriately trained and have access needs supported. Due to COVID-19, we may have to carry out meetings remotely. To ensure our opportunities are inclusive, we will add a phone number to the workshop advert and advertise through community groups. We will send guidance and pay for any dongles for those without internet access. Members will be paid £25/hour and £5 for any online interaction. We will evaluate the impact of our PPI using the GRIPP2 academic tool and asking for feedback.

10.4 Protocol compliance

Prospective, planned deviations or waivers to the protocol are not allowed under the UK regulations on Clinical Trials and must not be used.

Accidental protocol deviations must be adequately documented on the relevant forms and reported to the Chief Investigator and Sponsor immediately.

10.5 Monitoring and audit

The study may be subject to inspection and audit by Imperial College London under their remit as sponsor and other regulatory bodies to ensure adherence to GCP and the UK Policy Framework for Health and Social Care Research. The Chief Investigator will be responsible for the monitoring of the study.

10.6 Indemnity

- Given this is a data-only study, with no direct contact with participants, the potential legal liability of the sponsor(s) for harm to participants arising from the management, design, and conduct of the research is felt to be minimal.
- The study only involves sites that are covered by the NHS indemnity scheme.

- Imperial College holds Public Liability ("negligent harm") and Clinical Trial ("nonnegligent harm") insurance policies which apply to this trial. If a participant can demonstrate that they experienced harm or injury as a result of their participation in this trial, they will be eligible to claim compensation without having to prove that Imperial College is at fault. If the injury resulted from any procedure which is not part of the study, Imperial College will not be required to compensate them in this way. The participant's legal rights to claim compensation for injury where they can prove negligence are not affected.
- In addition, the study and protocol will be submitted to each participating site's Research & Development Department for the usual legal approvals.

10.7 Sponsor

Imperial College London will act as the main Sponsor for this study. Delegated responsibilities will be assigned to the NHS trusts taking part in this study.

10.8 Funding

NIHR Artificial Intelligence (AI Award 2020 Phase 3) Competition is funding the study. This includes funding to collect the datasets and run the studies at the NHS sites. Google will not receive grant funding from the AI Award, and will cover the costs of its staff, technology, and infrastructure.

10.9 Financial and other competing interests

Members of the study group will be required to declare:

- ownership interests that may be related to products, services, or interventions considered for use in the trial or that may be significantly affected by the trial,
- commercial ties requiring disclosure include, but are not restricted to, any pharmaceutical, behaviour modification, and/or technology company
- any non-commercial potential conflicts e.g. professional collaborations that may impact on academic promotion.
- A record of declarations will be kept by the Chief Investigator.

10.10 Amendments

- Amendments will be submitted to the relevant REC using forms provided by the HRA.
- The Chief Investigator will be responsible for the decision to amend the protocol and for deciding whether an amendment is substantial or non-substantial.
- Copies of amendment documentation will be provided to relevant stakeholders including R&D departments at each site.
- A record of changes will be maintained by the Chief Investigator.

10.11 Assessment and management of risk

Part A and B are studies using retrospective data >3 years old, and the algorithm will have no impact or influence on routine clinical care received by patients enrolled in this study. All women included in these studies will have had a subsequent screening round to qualify for inclusion, and so previously missed cancers are expected to be identified at this subsequent round. For this reason, the AI system and study is not considered to pose a direct risk to participants or the care they receive.

Ensuring the highest level of data security and privacy is a fundamental underlying theme of this project, as described in detail in Section 8. Great care will be taken to minimise any potential risk to patients due to data breach.

10.12 Potential for unintended bias

Google Health is very conscious of the potential for AI systems to propagate biases in healthcare. Blind spots in AI systems can reflect the worst societal biases, with a risk of unintended or unknown accuracies in minority subgroups. As a result, the AI system has been developed through the curation of large training datasets from two UK breast screening sites, encompassing scans of patients thought to closely represent the overall UK breast screening population. We believe that this methodology has minimised the possibility of algorithmic biases to date, and we have explored algorithm performance across many clinical and demographic subgroups.

Despite this, we need to achieve greater confidence about the AI system’s performance across various underrepresented subgroups that were not possible due to limited sample size in the original paper. Through the studies outlined in this application, we plan to more rigorously explore the performance of the system in larger, more diverse datasets, in order to ensure that any product created is safe for future deployment at scale.

Imperial College London

11 Expected outcomes of the study, Patient and Public Engagement and Dissemination

Through this project we aim to gather evidence of standalone accuracy from two large scale validations of the AI system, quantify human factors when being used by clinicians, and quantify workflow impacts and perform a full health economic assessment. For the next stage of this work, we plan to demonstrate feasibility of real world integration into live clinical systems. We hope this strategy will provide appropriate evidence of feasibility, efficacy and safety for the National Screening Committee to consider a future major modification to the screening programme. This work will also provide evidence for the wider clinical community to understand the impacts of this technology. This project seeks to understand factors that influence public engagement in AI technology, and what may foster better confidence in AI systems.

This study has clear anticipated public health benefits. Results from clinical, workflow and economic analyses will be used to design future interventional studies, and make recommendations about safe future integration of the AI system into routine clinical practice, supporting decision making by NHS England and the National Screening Committee. All results will also be presented at a formally designated Patient and Public Involvement and Engagement (PPIE) group whose interim and final meeting comments will be audited, presented to the steering committee and included in the final presentation of this work.

We plan for this work to be published in peer-reviewed academic journals with open source access as soon as possible following completion, following the forthcoming STARD-AI guidelines for diagnostic accuracy in AI studies (being led by members of this project (Sounderajah et al. 2020)). It is intended that the study will be published as a multicentre study. Findings will also be disseminated through infographics and blogs aimed at the general public, with the assistance of our patient and public representatives.

DocuSign Envelope ID: 8C37849F-C375-42F4-8310-CC1C11A57E2D

12 References

Breast Cancer Now. 2020. “Press Play: Getting and Keeping Breast Cancer Services Back on Track.” https://breastcancernow.org/sites/default/files/final_breast_cancer_now_press_play_report.pdf

DeLong, E. R., D. M. DeLong, and D. L. Clarke-Pearson. 1988. “Comparing the Areas under Two or More Correlated Receiver Operating Characteristic Curves: A Nonparametric Approach.” *Biometrics* 44 (3): 837–45.

Fagerland, Morten W., Stian Lydersen, and Petter Laake. 2014. “Recommended Tests and Confidence Intervals for Paired Binomial Proportions.” *Statistics in Medicine*. https://doi.org/10.1002/sim.6148.

Halling-Brown, Mark D., Lucy M. Warren, Dominic Ward, Emma Lewis, Alistair Mackenzie, Matthew G. Wallis, Louise S. Wilkinson, Rosalind M. Given-Wilson, Rita McAvinchey, and Kenneth C. Young. 2021. “OPTIMAM Mammography Image Database: A Large-Scale Resource of Mammography Images and Clinical Data.” *Radiology. Artificial Intelligence* 3 (1): e200103.

Liu, Jen-Pei, Huey-Miin Hsueh, Eric Hsieh, and James J. Chen. 2002. “Tests for Equivalence or Non-Inferiority for Paired Binary Data.” *Statistics in Medicine* 21 (2): 231–45.

Macmillan Cancer Support. 2020. “The Forgotten ‘C’? The Impact of Covid-19 on Cancer Care.” https://www.macmillan.org.uk/assets/forgotten-c-impact-of-covid-19-on-cancer-care.pdf.

Mann, H. B., and D. R. Whitney. 1947. *On a Test of Whether One of Two Random Variables Is Stochastically Larger Than the Other*.

McKinney, Scott Mayer, Marcin Sieniek, Varun Godbole, Jonathan Godwin, Natasha Antropova, Hutan Ashrafian, Trevor Back, et al. 2020. “International Evaluation of an AI System for Breast Cancer Screening.” *Nature* 577 (7788): 89–94.

Moser, Kath, Sarah Sellars, Margot Wheaton, Julie Cooke, Alison Duncan, Anthony Maxwell, Michael Michell, et al. 2011. “Extending the Age Range for Breast Screening in England: Pilot Study to Assess the Feasibility and Acceptability of Randomization.” *Journal of Medical Screening* 18 (2): 96–102.

Obuchowski, Nancy A. 1998. “On the Comparison of Correlated Proportions for Clustered Data.” *Statistics in Medicine*. https://doi.org/10.1002/(sici)1097-0258(19980715)17:13<1495::aid-sim863>3.0.co;2-i.

Public Health England. 2016. “NHS Breast Screening Programme: National Radiographic Workforce Survey 2016.” https://assets.publishing.service.gov.uk/government/uploads/system/uploads/attachment_data/file/564515/Final_radiographic_workforce_report_25-10-16_colinbabb_gateway_number_2016416.pdf.

Sounderajah, Viknesh, Hutan Ashrafian, Ravi Aggarwal, Jeffrey De Fauw, Alastair K. Denniston, Felix Greaves, Alan Karthikesalingam, et al. 2020. “Developing Specific Reporting Guidelines for Diagnostic Accuracy Studies Assessing AI Interventions: The STARD-AI Steering Group.” *Nature Medicine* 26 (6): 807–8.

The Royal College of Radiologists. 2021. “Clinical Radiology: UK Workforce Census 2020 Report.” https://www.rcr.ac.uk/system/files/publication/field_publication_files/clinical-radiology-uk-workforce-census-2020-report.pdf.

DocuSign Envelope ID: 8C37849F-C375-42F4-8310-CC1C11A57E2D

13 Appendix 1

RiViewer PACs-less, Vendor-Neutral Image Viewer

RiViewer is a bespoke software tool developed by the Royal Surrey NHS Foundation Trust which enables remote observer studies, collaborative viewing sessions and training. It is an application designed to allow workstation-independent, PACS-less viewing and interaction with de-identified medical images (e.g., for observer studies). RiViewer is a standalone tool designed to stream images from a cloud environment to a Radiology Workstation to allow study participants to view medical images, interact with them on studies and annotate ground truth.

Regions of interest (ROIs) can be identified by a user and any associated information about a mark, an image or a study can be added. The questions and settings can be easily configured by the researcher depending on the needs of the research. The extensible nature of the design allows for specific functionality and hanging protocols to be available for each study. Panning, windowing, zooming, and moving through slices are all available while modality-specific features can be easily enabled e.g., quadrant zooming in mammographic studies.

RiViewer is designed to stream images from cloud storage, allowing remote access by radiologists at any site with a workstation and internet connection. Due to the advanced workstation-style functionality, the simple deployment on heterogeneous systems over the internet RiViewer has been used for running remote paperless observer studies and can provide a training infrastructure and coordinating remote collaborative viewing sessions.

For Part B, RiViewer will be used to allow image readers at the clinical sites to define the ground truth on the images collected (locations of biopsy-proven lesions) and to view the images during both arms of the arbitration study. In the AI-enabled group, this will involve displaying the overlaid secondary capture objects outputted by the AI system (the location-based prediction).

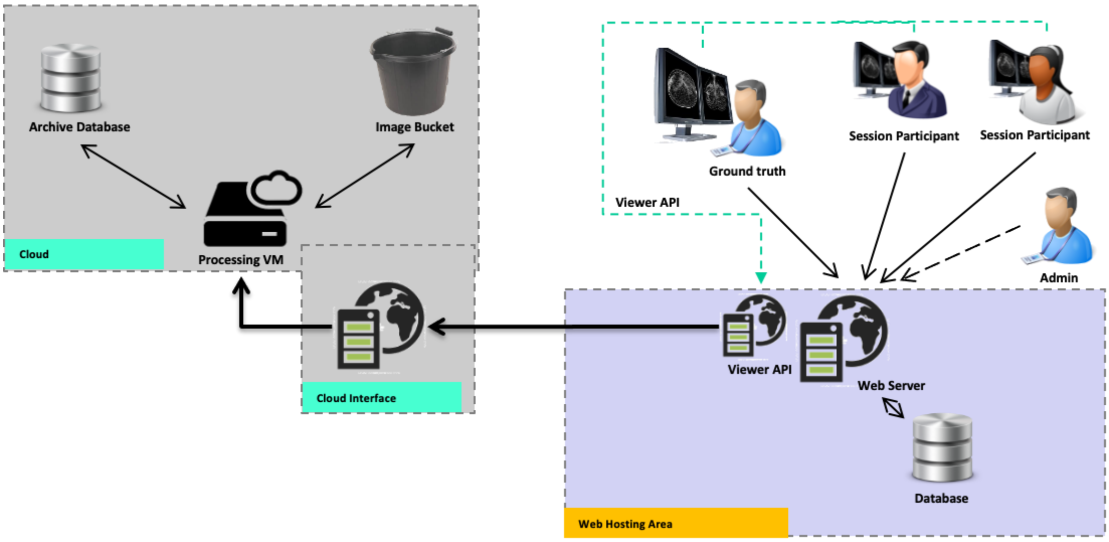

Figure 1: RiViewer Dataflows

DocuSign Envelope ID: 8C37849F-C375-42F4-8310-CC1C11A57E2D

RiViewer is a standalone executable, which launches a compiled Java program, bundled with its own Java Runtime Environment. This means that RiViewer does not need installation, does not create any system files, or interact with any system files. It runs within its own folder system. This makes it very simple to install on a workstation. The Zip file needs to be downloaded and extracted in a suitable location on the file system.

Reading sessions (for the arbitration study or ground truth) are prepared in the cloud. A user logs into RiViewer, which queries the cloud API to discover the reading session assigned to that user. The session properties file is downloaded which contains a list of the images that make up the session. RiViewer uses the user authentication tokens to request the images from the cloud and stream them to the local system. Users can view existing ROIs or overlays and are able to draw new ROIs and answer questions. The ROIs or questions answered are sent to the cloud API for storage in the reading sessions database.
