# Supplementary material for: Impact of using artificial intelligence as a second reader in breast screening including arbitration
Source: Nat Cancer. 2026 Mar 10;7(3):507–21. doi: 10.1038/s43018-026-01128-z (PMC13035470; doi:10.1038/s43018-026-01128-z)
Supplement: Supplementary file 2 — Reporting Summary [file 43018_2026_1128_MOESM2_ESM.pdf]

nature portfolio

Corresponding author(s): Lucy Warren, Hutan Ashrafian

Last updated by author(s): Nov 18, 2025

Reporting Summary

Nature Portfolio wishes to improve the reproducibility of the work that we publish. This form provides structure for consistency and transparency in reporting. For further information on Nature Portfolio policies, see our Editorial Policies and the Editorial Policy Checklist.

Statistics

For all statistical analyses, confirm that the following items are present in the figure legend, table legend, main text, or Methods section.

n/a	Confirmed	
☐	☒	The exact sample size (*n*) for each experimental group/condition, given as a discrete number and unit of measurement
☒	☐	A statement on whether measurements were taken from distinct samples or whether the same sample was measured repeatedly
☐	☒	The statistical test(s) used AND whether they are one- or two-sided<br>*Only common tests should be described solely by name; describe more complex techniques in the Methods section.*
☒	☐	A description of all covariates tested
☐	☒	A description of any assumptions or corrections, such as tests of normality and adjustment for multiple comparisons
☐	☒	A full description of the statistical parameters including central tendency (e.g. means) or other basic estimates (e.g. regression coefficient) AND variation (e.g. standard deviation) or associated estimates of uncertainty (e.g. confidence intervals)
☐	☒	For null hypothesis testing, the test statistic (e.g. *F*, *t*, *r*) with confidence intervals, effect sizes, degrees of freedom and *P* value noted<br>*Give P values as exact values whenever suitable.*
☒	☐	For Bayesian analysis, information on the choice of priors and Markov chain Monte Carlo settings
☒	☐	For hierarchical and complex designs, identification of the appropriate level for tests and full reporting of outcomes
☒	☐	Estimates of effect sizes (e.g. Cohen's *d*, Pearson's *r*), indicating how they were calculated

Our web collection on statistics for biologists contains articles on many of the points above.

Software and code

Policy information about availability of computer code

Data collection	The AI tool (v1.2, Google LLC) was not developed in this study, it was evaluated. The code used for training the models has a large number of dependencies on internal tooling, infrastructure and hardware, and its release is therefore not feasible. However, implementation details are described in sufficient detail in the Supplementary Methods of McKinney et al. (8), and Kelly et al. (9) to support replication with non-proprietary libraries.The AI system is not currently available for external use but interested parties should contact the corresponding authors of the relevant paper (9) to be notified about external availability.
Data analysis	Statistical analysis and plotting was performed using open source and publicly available Python packages (numpy v1.24.4, pandas v2.1.1, scipy v1.9.1, matplotlib v3.7.2, seaborn v0.13.0), using the methodology outlined in the Methods section. All codes were executed in Python (v.3.9.7).

For manuscripts utilizing custom algorithms or software that are central to the research but not yet described in published literature, software must be made available to editors and reviewers. We strongly encourage code deposition in a community repository (e.g. GitHub). See the Nature Portfolio guidelines for submitting code & software for further information.

Data

Policy information about availability of data

All manuscripts must include a data availability statement. This statement should provide the following information, where applicable:

- Accession codes, unique identifiers, or web links for publicly available datasets
- A description of any restrictions on data availability
- For clinical datasets or third party data, please ensure that the statement adheres to our policy

The images and clinical data used in this publication are from the OPTIMAM imaging database and are not publicly available due to restrictions imposed by OPTIMAMs ethical approval. Instead, the images and data can be accessed after a formal data access request and review by a Data Access Committee and a Data Sharing Agreement (DSA) implemented. Applications for access to the data can be made at https://medphys.royalsurrey.nhs.uk/omidb/getting-access/. The application, review and agreement process can take anywhere from 2-12 weeks depending upon the applicants desire to customise the template DSA. The dataset derived from this resource that supports the primary findings of this study is available in Supplemental Table 4. Source data for Fig. 1e, 2a-d, 3 and 4a-d have been provided as Source Data files.

Research involving human participants, their data, or biological material

Policy information about studies with human participants or human data. See also policy information about sex, gender (identity/presentation), and sexual orientation and race, ethnicity and racism.

Reporting on sex and gender	This study is focused on population breast cancer screening - in the UK females aged 50-70 years are invited to screening. Throughout the paper we refer to the population as women, which is what is also used in the information leaflets for the breast screening programme https://assets.publishing.service.gov.uk/media/659fd32de8f5ec000f1f8ba8/BSP01_Dec23.pdf .
Reporting on race, ethnicity, or other socially relevant groupings	We report subgroup analyses on ethnicity (as defined by the UK government - https://www.ethnicity-facts-figures.service.gov.uk/style-guide/ethnic-groups/) and rely on routinely collected NHS data for these analyses. We also analyse results using Indices of Multiple Deprivation as a measure of socioeconomic status (https://data.cdrc.ac.uk/dataset/index-multiple-deprivation-imd) and calculate this using LSOA data prior to anonymisation.
Population characteristics	50,000 women, aged 50-70, from two screening centres (25,000 each centre). The subgroups considered related to population were breast density category, age, type of screen (first or subsequent), index of multiple deprivation and ethnicity.
Recruitment	Participants were not recruited in this study, this was a retrospective study, and the data was from the OPTIMAM imaging database (https://pubs.rsna.org/doi/full/10.1148/ryai.2020200103)
Ethics oversight	The study protocol was approved by East Midlands Nottingham Research Ethics Committee (22/EM/0038) and NHS England Breast Screening Programme Research Advisory Committee (BSPRAC_0093).

Note that full information on the approval of the study protocol must also be provided in the manuscript.

Field-specific reporting

Please select the one below that is the best fit for your research. If you are not sure, read the appropriate sections before making your selection.

☒ Life sciences ☐ Behavioural & social sciences ☐ Ecological, evolutionary & environmental sciences

For a reference copy of the document with all sections, see nature.com/documents/nr-reporting-summary-flat.pdf

Life sciences study design

All studies must disclose on these points even when the disclosure is negative.

Sample size	We powered the study by simulating a two-arm, within-case design (routine vs AI-assisted), where each case is read under both regimes and where the primary analysis is a matched-pair Wald test for non-inferiority on sensitivity (specificity was expected to be amply powered given low prevalence). We assumed identical underlying performance in both arms: latent continuous scores with AUC = 0.90, binarised at a common threshold to yield 73% sensitivity and specificity using 39-month outcomes. Between-arm correlation was modelled via an agreement parameter set to 84.5%, matching previously observed R1–R2 concordance on positives. We modelled the two site-specific arbitration regimes (R1|R2 and R1≠R2) and powered the study using a worst case scenario that combined the R1|R2 arbitration style, consensus panel recall = 0.73 and agreement between arms = 0.70. Under these assumptions, 275 cancer-positive cases exceeded 90% power, while 200 positives provided 80% power. We therefore targeted a minimum of 200 positive cases per site to achieve 80% power. Assuming a population prevalence of cancer, this corresponded to 25,000 cases per site.
Data exclusions	The exclusions were defined prior to the study. From the 50,000 women, 4,354 women (8.7%) were excluded due to being within the AI exclusion criteria (technical recalls, cases containing more or less than 4 images, and implants) and 44 (0.1%) cases were excluded due to insufficient or conflicting clinical information.
Replication	This was not applicable to this study, as it was a retrospective study and all clients were in both arms of the study.

Randomization	This was not applicable to this study, as it was a retrospective study and all clients were in both arms of the study.
Blinding	It was not possible to blind the arm to the readers as the AI output was overlaid on images, and the human readers' decisions on paperwork. However, this is clinically realistic as it is how the images would be read clinically.

Reporting for specific materials, systems and methods

We require information from authors about some types of materials, experimental systems and methods used in many studies. Here, indicate whether each material, system or method listed is relevant to your study. If you are not sure if a list item applies to your research, read the appropriate section before selecting a response.

Materials & experimental systems

n/a	Involved in the study
☒	☐ Antibodies
☒	☐ Eukaryotic cell lines
☒	☐ Palaeontology and archaeology
☒	☐ Animals and other organisms
☐	☒ Clinical data
☒	☐ Dual use research of concern
☒	☐ Plants

Methods

n/a	Involved in the study
☒	☐ ChIP-seq
☒	☐ Flow cytometry
☒	☐ MRI-based neuroimaging

Clinical data

Policy information about clinical studies

All manuscripts should comply with the ICMJE guidelines for publication of clinical research and a completed CONSORT checklist must be included with all submissions.

Clinical trial registration	The study was registered with ISRCTN (60839016)
Study protocol	The study protocol has been provided in the supplemental information.
Data collection	Mammography images and clinical data for 50,000 women from two NHSBSP screening centres in London were selected from the OPTIMAM Mammography Image Database OMI-DB (https://pubs.rsna.org/doi/full/10.1148/ryai.2020200103). We selected a random selection of 25,000 women from each screening centre from 2016, allowing for 3 years follow up at the next screening round. Women aged 67 or below with normal mammograms without 3 years' follow-up were replaced with women with 3 year follow-up examination, matched by episode outcome, whether it was first or subsequent screening and age. Women aged 68+ were permitted to have no follow up screen, as they would not typically be invited back as part of national screening at this age. For one Screening Centre, there was also a proportion of women whose cases had been used to train the AI tool. These were also replaced with women who had not been used to train the AI tool.
Outcomes	Primary outcome measure was pre-defined as non-inferiority (prespecified 5% absolute margin) of the AI Arm for sensitivity and specificity at the case level, compared to Human Arm, measured against a 39-month ground truth.<br><br>Secondary outcome measures were pre-defined as positive predictive value, negative predictive value, cancer detection rate and recall rate.<br><br>Exploratory outcome measures were pre-defined as subgroups analysis by type of screen (first or subsequent), age, ethnicity, X-ray system manufacturer index of multiple deprivation, breast density, cancer type, cancer grade, conspicuity, lesion characteristic and lesion size. In addition, localisation analysis of the bounding boxes drawn during arbitration.

Plants

Seed stocks	*Report on the source of all seed stocks or other plant material used. If applicable, state the seed stock centre and catalogue number. If plant specimens were collected from the field, describe the collection location, date and sampling procedures.*
Novel plant genotypes	*Describe the methods by which all novel plant genotypes were produced. This includes those generated by transgenic approaches, gene editing, chemical/radiation-based mutagenesis and hybridization. For transgenic lines, describe the transformation method, the number of independent lines analyzed and the generation upon which experiments were performed. For gene-edited lines, describe the editor used, the endogenous sequence targeted for editing, the targeting guide RNA sequence (if applicable) and how the editor was applied.*
Authentication	*Describe any authentication procedures for each seed stock used or novel genotype generated. Describe any experiments used to assess the effect of a mutation and, where applicable, how potential secondary effects (e.g. second site T-DNA insertions, mosiacism, off-target gene editing) were examined.*
